# Ce6-modified Fe ions-doped carbon dots as multifunctional nanoplatform for ferroptosis and photodynamic synergistic therapy of melanoma

**DOI:** 10.1186/s12951-024-02346-2

**Published:** 2024-03-10

**Authors:** Haiqiu Li, Yichen Dou, Hang Yang, Hanlin Xing, Cheng Zhu, Tao Wang, Zhaopeng Xuan, Mingxi Yang

**Affiliations:** 1 Department of Hand and Foot Surgery, Orthopedics Center, The First Hospital of Jilin University, Jilin University, Changchun, 130031 People’s Republic of China; 2 Department of Anesthesiology, The First Hospital of Jilin University, Jilin University, Changchun, 130031 People’s Republic of China; 3 Joint Laboratory of Opto-Functional Theranostics in Medicine and Chemistry, The First Hospital of Jilin University, Changchun, 130021 People’s Republic of China

**Keywords:** Carbon dots, Fe ions-doping, Ce6, Ferroptosis, Photodynamic therapy, Melanoma

## Abstract

**Background:**

Despite the higher sensitivity of melanoma towards ferroptosis and photodynamic therapy (PDT), the lack of efficient ferroptosis inducers and the poor solubility of photosensitizers restrict their synergistic strategies. With unique advantages, carbon dots (CDs) are expected to serve as innovative building blocks for combination therapy of cancers.

**Results:**

Herein, an ferroptosis/PDT integrated nanoplatform for melanoma therapy is constructed based on chlorin e6-modified Fe ions-doped carbon dots (Fe-CDs@Ce6). As a novel type of iron-carbon hybrid nanoparticles, the as-prepared Fe-CDs can selectively activate ferroptosis, prevent angiogenesis and inhibit the migration of mouse skin melanoma cells (B16), but have no toxicity to normal cells. The nano-conjugated structures facilitate not only the aqueous dispersibility of Ce6, but also the self-accumulation ability of Fe-CDs@Ce6 within melanoma area without requiring extra targets. Moreover, the therapeutic effects of Fe-CDs@Ce6 are synergistically enhanced due to the increased GSH depletion by PDT and the elevated singlet oxygen (^1^O_2_) production efficiency by Fe-CDs. When combined with laser irradiation, the tumor growth can be significantly suppressed by Fe-CDs@Ce6 through cyclic administration. The *T*_*2*_-weighted magnetic resonance imaging (MRI) capability of Fe-CDs@Ce6 also reveals their potentials for cancer diagnosis and navigation therapy.

**Conclusions:**

Our findings indicate the multifunctionality of Fe-CDs@Ce6 in effectively combining ferroptosis/PDT therapy, tumor targeting and MRI imaging, which enables Fe-CDs@Ce6 to become promising biocompatible nanoplatform for the treatment of melanoma.

**Graphic Abstract:**

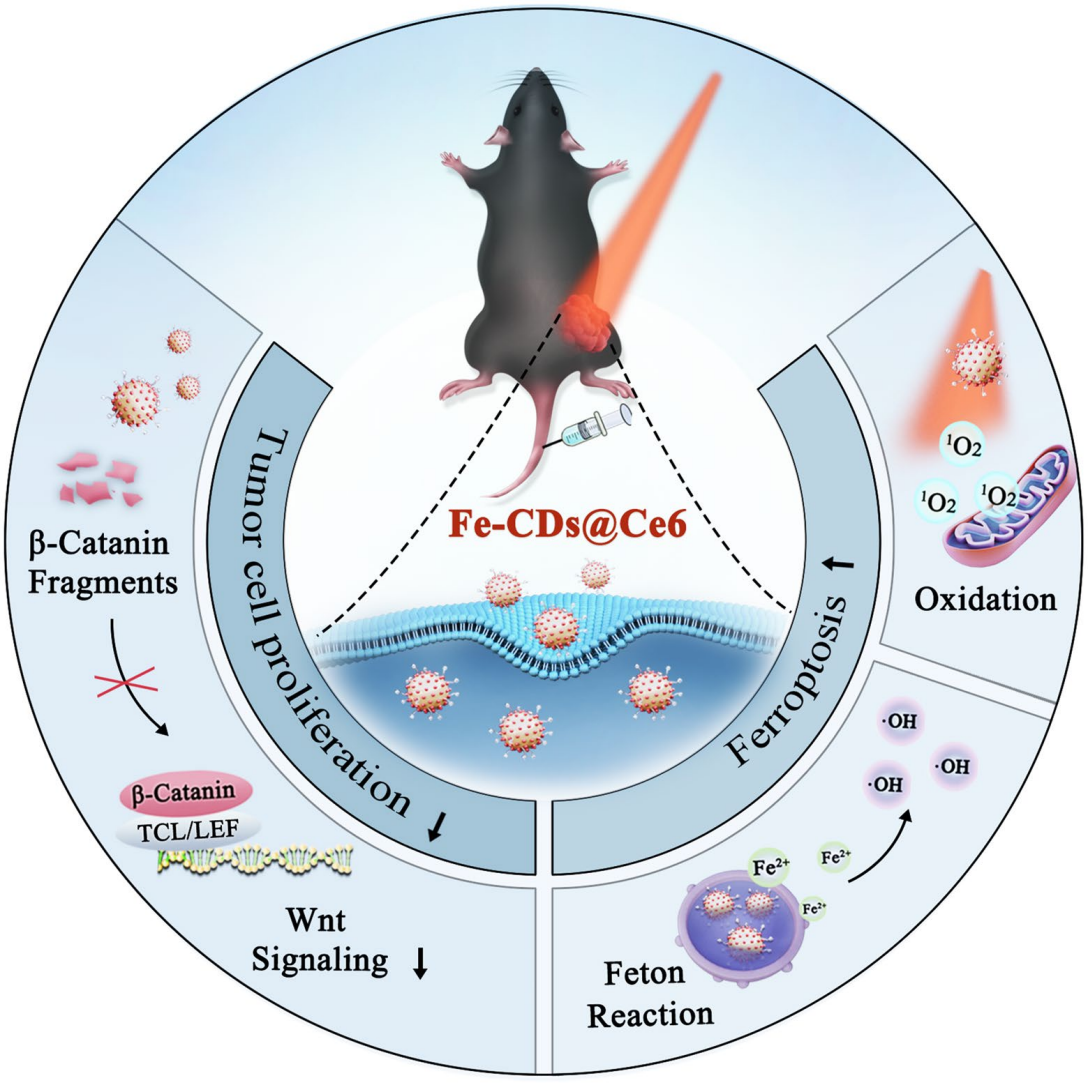

**Supplementary Information:**

The online version contains supplementary material available at 10.1186/s12951-024-02346-2.

## Introduction

Melanoma is a highly invasive malignant tumor, with 5-year survival rate below 30% once metastasized, accounting for the majority of deaths from skin cancer [1–3]. The extremely strong drug resistance and relapse trend lead to the insensitivity of melanoma towards chemotherapy, radiotherapy and immunotherapy, as well as failed rehabilitation after surgical treatment [4, 5]. Ferroptosis is a form of iron-dependent programmed cell death that involves the processes such as accumulation of intracellular iron, depletion of antioxidant defenses, and lipid peroxidation [6, 7]. The management of iron ions, predominantly in the form of redox-active Fe^2+^, plays a pivotal role in facilitating the elevation of reactive oxygen species (ROS) through mechanism such as Fenton reactions and glutathione (GSH) consumption [8, 9]. The concept of ferroptosis has provided innovative insights into the development of effective combination therapies and synergistic strategies for cancer treatments [10–12]. According to previous reports, melanoma is potentially more susceptible to ferroptosis than other tumors, which stems from the higher levels of dissociative iron in melanoma cells [13], revealing the bright prospect of ferroptosis in developing innovative strategies for melanoma therapy. Nevertheless, there is still a lack of stable and valid ferroptosis inducers, highlighting the need for further optimization of novel nanomaterials to activate ferroptosis.

Over the past decades, carbon dots (CDs), an emerging type of carbon nanomaterials, have revealed bright prospects in the field of drug delivery and disease therapy [14–17]. The prominent advantages of CDs, such as ultra-small size, exceptional biosafety, modifiable and stable structures, make it an ideal alternative for designing and mimicking nanozymes in biological systems [18–20]. Recently, CDs derived from natural product, i.e., Ginseng [21] and coffee extract [22], have been reported to exhibit GSH oxidase-like activities, which can enhance ferroptosis-dependent anti-tumor immunity [23]. However, despite the fact that GSH oxidase-like enzymes can disrupt the glutathione peroxidase 4 (GPX4)-catalyzed lipid peroxidation repair system and promote the vulnerability of cells towards ferroptosis, they are generally incapable of directly inducing strong ferroptosis effects in tumor cells [24, 25]. In biological systems, the regulation of iron level is accomplished via transferrin-mediated iron uptake and the autophagic/lysosomal degradation of endogenous ferritin [26–30]. Considering that the above mentioned iron-containing proteins and other iron oxide nanoparticles [31, 32] are often restricted by their limited effect, low selectivity, and poor stability, the development of iron-carrying nanoagents based on CDs may have innovative significance in ferroptosis research.

In addition, the adjacent lymphatic microenvironment allows cellular infiltration of antioxidants, which provides the possibility of ferroptosis escape and resistance for melanoma cells. Photodynamic therapy (PDT), that involves the generation of reactive oxygen species (ROS) by photosensitizers (PS) under laser excitation [33–36], has been reported to accelerate the destruction of oxidative homeostasis and enhance the effect of ferroptosis [37]. Particularly, Fe-induced Fenton reaction can also compensate for the hypoxic environment in tumor cells, conversely strengthening PDT efficacy, resulting in a synergistic amplification of anti-tumor capability [38, 39]. However, traditional PS chlorin e6 (Ce6) suffers from several drawbacks such as poor solubility, lack of tumor targeting. Although various delivery systems are reported for encapsulation and transportation of Ce6, the difficulties in metabolism and relatively large size of the carriers still limit their further application. Fortunately, the abundant surface functional groups and the nano-size effect endow CDs with unique merits as Ce6 delivery systems [40–44]. To the best of our knowledge, there are no reports on CDs-based ferroptosis/PDT synergistic therapy nanoplatform for the treatment of melanoma.

**Scheme 1:**
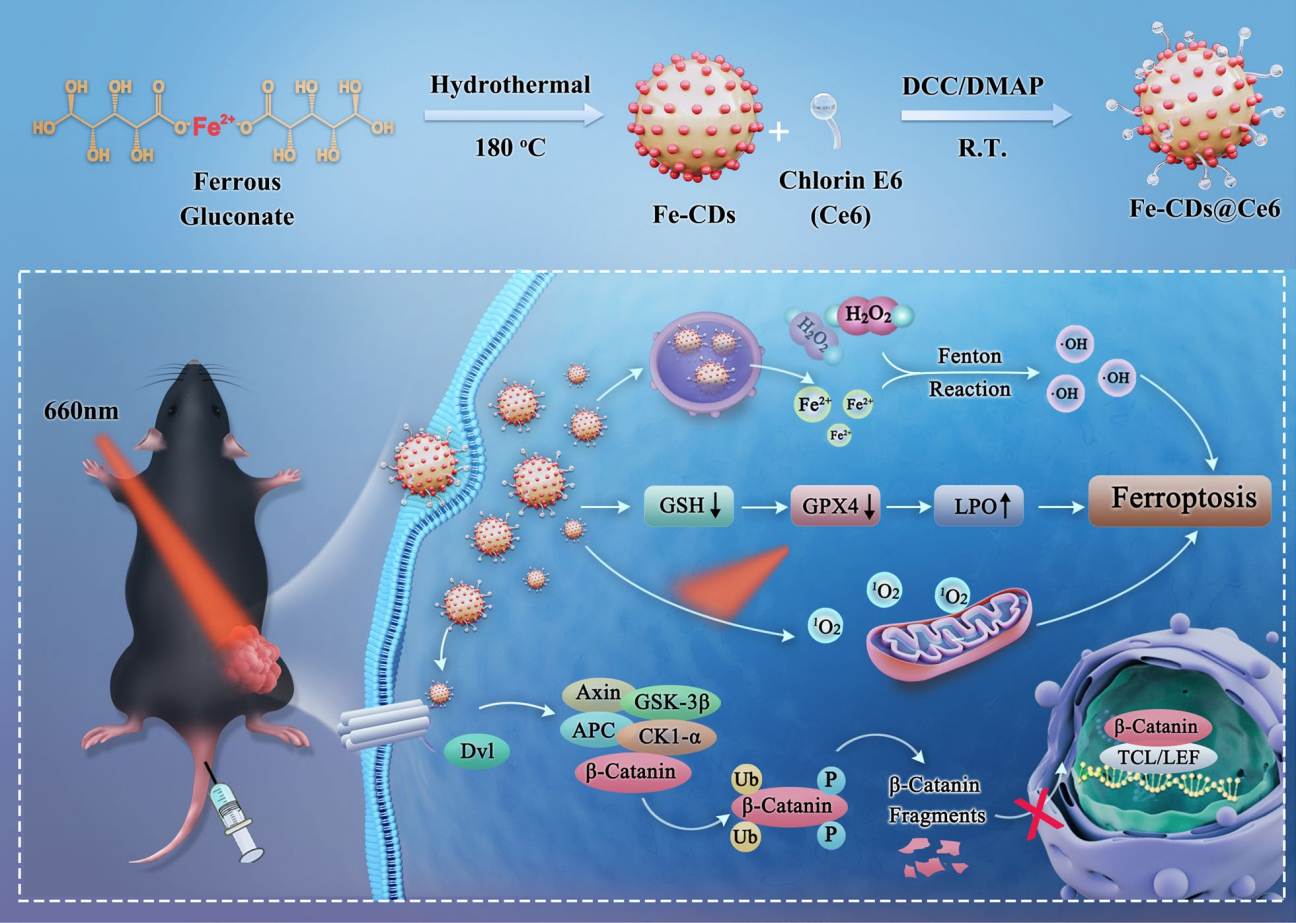
Schematic illustration of the synthesis procedure of Ce6-modified Fe ions-doped carbon dots (Fe-CDs@Ce6) as a multifunctional nanoplatform for ferroptosis/photodynamic synergistic melanoma therapy

Inspired by this, the present work first report the synthesis of biocompatible Fe ions-doped CDs (Fe-CDs) using clinically approved nutritional iron supplements. The oxidized Fe ions in Fe-CDs can be transformed into Fe^2+^ in response to the high GSH level in tumor cells. Therefore, Fe-CDs can effectively initiate the ferroptosis pathway, induce the apoptosis of melanoma cells and inhibit migration, with no significant toxicity to normal cells. Afterwards, a novel type of Fe-CDs@Ce6 nanoplatform is developed with multifunction of ferroptosis/PDT synergistic therapy and magnetic resonance imaging (MRI). This integrated approach not only enhances the therapeutic efficacy of Fe-CDs, but also contributes to intervening in the tumor microenvironment and limiting the invasive and metastatic capabilities of melanoma cells, thereby providing a more comprehensive treatment outcome. Through cyclic administration, the nanoconjugates of Fe-CDs@Ce6 accumulate in the dorsal melanoma area of mice, and the melanoma are almost ablated when introduced 660 nm laser irradiation. Moreover, the tumor metastasis and recurrence are also suppressed due to anti-tumor immune activation and angiogenesis inhibition (Scheme 1). This study proposes a nanosystem that combines ferroptosis and PDT treatment to improve the solubility and tumor enrichment of photosensitizers, thus reduce the side effects and enhance the therapeutic efficacy for tumor therapy.

## Methods

### Materials

Ferrous gluconate hydrate (GlcFe) and chlorin e6 (Ce6) were purchased from Macklin Reagent Inc. (Shanghai, China). *N*,*N*′-Dicyclohexylcarbodiimide (DCC), 4-dimethylaminopyridine (DMAP), and dimethyl sulfoxide (DMSO) were purchased from Aladdin Industries Inc. (Shanghai, China). All the reagents were used directly without further purification. Deionized (DI) water was used throughout the whole experiments.

### Synthesis of Fe-CDs

The Fe ions-doped carbon dots (Fe-CDs) were prepared based on our previous work with some modification [45]. Briefly, 964.34 mg (2 mmol) GlcFe were added in 40 mL DI water and completely dissolved by magnetic stirring for 5 min. The solution was poured into a 100 mL Teflon-lined autoclave and enclosed within a matched stainless steel reactor. Then, the reactor was placed in a heating oven and subjected to hydrothermal reaction at 180 ℃ for 4 h. After which, the kettle was taken out from the oven and cooled naturally to room temperature. The reaction solution was then centrifuged at 8000 rpm for 10 min to remove aggregated particles and insoluble precipitates. The supernatant was further filtered through a 0.22 μm polyethersulfone membrane, followed by purification for at least 2 d in a 500–1000 Da molecular weight cut-off cellulose ester (CE) dialysis bag to remove raw materials and small molecular fragments. Finally, pure powder of Fe-CDs could be obtained by desiccated in a vacuum freeze dryer.

### Synthesis of Fe-CDs@Ce6

The Ce6-modified Fe-CDs (Fe-CDs@Ce6) was synthesized by catalyzed esterification coupling reaction. Generally, a certain amount of Ce6 was weighed and dissolved in DMSO at a concentration of 2 mg/mL. Then, DCC with a molar mass of about twice that of Ce6 was added and stirred for 30 min to activate the carboxyl groups of Ce6. The working solution of Fe-CDs (2 mg/mL) was configured and mixed with the DMSO solution of Ce6 in a volume ratio of 2:1 under stirring. Afterwards, DMAP with equal molar mass of DCC was added into the mixture and the coupling reaction was proceeded at room temperature for 12 h. The solution was dialyzed in a 500–1000 Da CE dialysis bag for 4 d to completely remove the unreacted free molecules and DMSO solvent. The solution of Fe-CDs@Ce6 was concentrated by rotary evaporation, and then a certain volume of the solution was dried and weighed to determine the concentration.

### Characterization

Transmission electron microscope (TEM) images were photographed on JEOL JEM-2100F. Photoluminescence (PL) spectra was recorded on Shimadzu RF-5301 PC. X-ray photoelectron spectroscopy (XPS) analysis was measured from ESCALAB 250 spectrometer with a mono X-ray source Al Kα excitation (1486.6 eV). Fourier transformed infrared spectra (FTIR) was measured on Nicolet Avatar 360 FTIR spectrophotometer. Ultraviolet–Visible (UV–Vis) absorption spectra was recorded on Shimadzu 3100 UV–Vis spectrophotometer. The metal content of Fe-CDs was determined by Agilent 725 inductively coupled plasma (ICP) spectrometer.

### Cell culture

The mouse melanoma cell line (B16, TCM2, Shanghai, China) were provided by Chinese Academy of Sciences Cell Bank. B16 were cultured in an incubator with RPMI-1640 (11875093, Gibco, USA), 1% antibiotic antifungal preparations (C100C5, NCM, China) and 10% fetal bovine serum (FBS, FB25015, Clark, Australia) at 37 ℃ and 5% CO_2_, and were gathered when the cells reached 80–90% confluence. The human umbilical vein endothelial cells (HUVECs) were purchased from Fuheng Biotechnology Co., Ltd. (FH1122, Shanghai, China). HUVECs were cultured in endothelial cell medium (ECM, ScienCell) with 10% FBS and endothelial growth medium supplements. The rat Schwann cells (RSC96) were purchased from Chinese Academy of Sciences Cell Bank (GNR6, Shanghai, China). RSC96 were cultured in Dulbecco’s modified eagle medium (DMEM, 03.1002C, EallBio, China) with 10% FBS. All the cells were incubated in a humidified incubator (37 ℃, 5% CO_2_), and checked regularly for mycoplasma contamination. They were detached from the growth surface using a trypsin/EDTA solution (C100C1, NCM, China) prior to analysis, and maintained in logarithmic growth during all biological assays.

### Cytotoxicity assay

Cytotoxicity assays using Cell Counting Kit-8 (C0038, Beyotime, China) were conducted for measuring the cytotoxicity of Fe-CDs. First, Fe-CDs was diluted to 50, 100, 200, 300, 400, and 500 μg/mL. Then, CCK-8 assays were performed to determine the cell viability of B16, RSC96 and HUVECs according to manufacturer’s instructions. The absorbance at 450 nm was detected using a multidetection microplate reader (BioTek microplate reader, USA). The experimental process was repeated three times in order to remove operating error.

### In vitro experiments of anti-tumor and photodynamic effects

#### Living/dead assay

The cells were cultured in RPMI-1640 containing 10% FBS and added with Ce6, Fe-CDs, Fe-CDs@Ce6 (200 µg/mL) and an equal volume of PBS (03.15018C, EallBio, China). A portable 660 nm laser with a power density of 100 mW/cm^2^ was irradiated onto Fe-CDs@Ce6 well for 10 min at the same distance (~ 8–10 cm) to provide the same laser power. After 24 h, Calcein-AM/PI Double Staining Kit (C2015M, Beyotime, China) was performed to measure the percentages of live (calcein-AM+, PI−) and dead (calcein-AM−, PI+) cells according to manufacturer’s instructions. The cell culture dish was observed and photographed under laser confocal microscope (Olympus FV1000).

#### Cell apoptosis assay

The cells were treated with PBS, Ce6 (80 µg/mL), Fe-CDs (200 µg/mL), Fe-CDs@Ce6 (200 µg/mL) and then apoptosis was detected with the KeyGEN FITC Annexin V/PI Apoptosis Detection Kit (C1062M, Beyotime, China). Briefly, cells were simultaneously stained with annexin V-FITC and PI, and incubated for 15 min at room temperature in 1 × annexin binding buffer. Then, the data from flow cytometry was analyzed using Flow Jo software (FlowJo LLC).

#### Cell colony assay

Cell colony formation ability was measured by plate colony formation assay. About 200 cells were added to each well of a 6-well plate and incubated for about 2 weeks. Meanwhile, the cells were treated with PBS, Ce6, Fe-CDs, Fe-CDs@Ce6 (200 µg/mL) until a colony was obviously formed. Then the plate was gently washed with PBS and stained with crystal violet (C0121, Beyotime, China). The number of colonies was determined by observing the proliferation of a single cell under an optical microscope.

#### Transwell invasion assay

Matrigel-coated Transwell chambers (CLS3422, Corning Transwell, USA) was used for Transwell migration assay. Transwell inserts with 8 µm pores were coated with Matrigel (E1270, Corning, USA) and reconstituted with fresh medium for 2 h before the experiment. B16 cells were seeded into the upper chambers with 500 μL of serum-free RPMI-1640 in 12-well plates, while 650 μL of RPMI-1640 supplemented with 10% FBS was placed in the lower chamber. After 24 h, the cells that migrated to the lower surface of the Matrigel-coated membrane were fixed and stained with crystal violet and counted.

#### Cell comet assay

The cells were intervened once after being treated with PBS, Ce6, Fe-CDs, and Fe-CDs@Ce6 (200 µg/mL) 24 h prior to collection. Comet assays were performed using the Comet Assay kit (4250-050-K, Trevigen, USA) according to manufacturer’s instructions. Briefly, the cells were washed once in ice-cold PBS, resuspended to 1 × 10^5^ cells/mL in PBS, mixed with molten low-melt agarose at the ratio of 1:10 (10/100 μL), spread onto Comet slides, allowed to set at 4 ℃ for 10 min, and lysed in Lysis Solution for 1–2 h. For alkaline comet assay, slides were transferred to Alkaline Unwinding Solution for 20 min and subjected to electrophoresis in Alkaline Electrophoresis Solution at 21 V for 30 min at 4 ℃. The slides were washed in DI water twice for 5 min, and immersed in 70% ethanol for 5 min, then dried at 37 ℃ for 15 min. The dried slides were stained with a 1:30,000 dilution of SYBR-green and the comet tail moment was quantified using the ImageJ plugin OpenComet.

#### Cell proliferation assay

The cells were co-incubated with EdU working solution (C0071S, Beyotime, Shanghai, China) for 2 h at 5% CO_2_ and 37 °C. Subsequently, EdU fluorescence labeling was performed using Alexa Fluor 488 (Beyotime) with the EdU Cell Proliferation Kit, while cell nuclei were stained with Hoechst solution (C1025, Beyotime) at room temperature in the dark. Proliferating cells were labeled in green, while the nuclei of all cells were stained blue. The images of EdU-positive cells were captured at 100 × magnification using an inverted fluorescence microscope (Olympus), and cell counting was carried out using ImageJ software. The percentage of EdU-positive cells was calculated using the following formula:

$${\text{EdU - positive rate = }}\left( {\frac{{\text{EdU - positive cell count}}}{{\text{EdU - positive cell count + EdU - negative cell count}}}} \right) \times 100\% {.}$$

### Detection of GSH level and singlet oxygen production

The level of GSH/GSSH was detected using the GSH/GSSG assay kit (Beyotime, Shanghai, China). The detection principle was that GSH reacted with the chromogenic substrate DTNB to produce yellow TNB. The amount of total cellular GSH can be calculated by detecting the absorbance at 412 nm with a microplate reader. After Ce6, Fe-CDs, Fe-CDs@Ce6, and Fe-CDs@Ce6 + PDT treatments, B16 cells were washed with PBS, recentrifuged at 1000*g* for 3 min, and the supernatant was aspirated. The cells were lysed by two freeze–thaw cycles in liquid nitrogen and 37 °C water bath. The molar concentrations of GSH and GSSG were calculated according to the standard curve, and the GSH contents were determined as the protein content per unit weight.

The generation of singlet oxygen (^1^O_2_) in vitro was detected with a singlet oxygen detection kit (S36002, Thermo fisher, USA). Briefly, 25 µL of 50 µM SOSG was added into the solutions containing free Fe-CDs (200 µg/mL), Ce6 (80 µg/mL), and Fe-CDs@Ce6 (200 µg/mL), followed by 660 nm laser irradiation at 100 mW/cm^2^ for different time periods (0, 10, 20, 30, 40, 50, 60 min). Subsequently, oxidized SOSG was detected by a microplate reader to represent ^1^O_2_ production. The blank aqueous solution was used as a negative control.

### Cellular ROS production and mitochondrial membrane potential assessment

Reactive oxygen species (ROS) levels in cells were measured using an ROS assay kit (S0033S, Beyotime, China). Briefly, after treating B16 cells with 200 μg/mL Fe-CDs, the culture medium was collected, and fresh medium was replaced. Subsequently, the collected medium containing B16-conditioned medium was used to culture HUVECs for 6 h before replacing it with fresh medium. Subsequently, the cells from the treated and control groups were incubated under DCFH-DA detection reagent (20 min, 5% CO_2_, 37 °C). After that, the cells were imaged at five different locations (four corners and one in the center, covering ~ 5/9 of the entire structure) using confocal microscope. Subsequently, the images were analyzed using ImageJ software to determine the fluorescence intensity directly associated with ROS levels.

The mitochondrial membrane potential was assessed using the JC-1 Mitochondrial Membrane Potential Assay Kit (C2006, Beyotime, China). After culturing B16 cells treated with Fe-CDs (200 μg/mL) and HUVECs cultured in conditioned medium from Fe-CDs-treated B16 cells for 6 h, the culture medium was replaced with fresh medium. The cells were then incubated with JC-1 working solution in a CO_2_ incubator for 20 min. After incubation, the staining solution was removed, and the cells were washed twice with JC-1 staining buffer. The cells were imaged using a confocal microscope, and changes of mitochondrial membrane potential (Δψm) were reflected by the ratio of green fluorescence to red fluorescence (ratio of JC-1 monomers to JC-1 aggregates).

### Stress fiber polymerization assay

After treating B16 cells with Fe-CDs (200 μg/mL) for 6 h, the cells were fixed in 4% paraformaldehyde for 30 min, permeabilized with 0.5% Triton X-100 (P0096, Beyotime, China) for 30 min, and blocked with 3% skimmed milk for 1 h. After each step, the cells were gently washed three times with PBS. Subsequently, B16 cells were stained for cellular cytoskeletal protein F-actin using fluorescein isothiocyanate (FITC)-labeled phalloidin (CA1620, Solarbio, China). The staining was observed under a laser confocal microscope (Olympus FV1000).

### Tube formation assay

To investigate the impact of Fe-CDs on angiogenesis, Matrigel tube formation assays were conducted using HUVECs following the manufacturer’s protocol (BD, Endothelial Cell Tube Formation assay). In brief, HUVECs were cultured with the conditioned medium from B16 cells previously treated with Fe-CDs (200 μg/mL) for 6 h. Matrigel (50 μL) was coated onto a 96-well culture plate and allowed to solidify at 37 °C for 1 h. Subsequently, the HUVECs were incubated at 37 °C for 2 h. The images of tube formation were captured using a microscope between 4 to 12 h.

### Intracellular distribution of Fe-CDs

B16 cells were cultured in RPMI-1640 containing 10% FBS and added with Fe-CDs and an equal volume of PBS. After 6 h, the cells were fixed with glutaraldehyde solution. Intracellular distribution of Fe-CDs was determined under transmission electron microscopy (80 kV, Philips EM208 or 120 kV, Tecnai12). The images were photographed at a magnification of × 50,000 or × 52,000, and scanned at 6 μm per pixel using an MRC-KZA scanner. High-resolution scanning electron microscope (FESEM, Merlin) demonstrated the intracellular distribution of Fe-CDs.

According to the manufacturer’s instructions, the Perl’s Prussian Blue Staining Kit (G1426, Solarbio, USA) was used to detect the distribution of intracellular iron ions. Briefly, the cells were fixed in 4% paraformaldehyde for 15 min, followed by incubation with 10% potassium ferrocyanide for 25–30 min, and then washed and restained with nuclear fast red for 10 min. The cells containing blue granules in the cytoplasm were considered positive for Prussian Blue staining.

### In vivo anti-tumor experiments and photodynamic effect

Male C57BL/6 mice (18–20 g of weight, 8 weeks of age) were obtained from Beijing Weitong Lihua Biotechnology Co., Ltd. The animals were housed in a well-equipped animal facility with an ambient temperature of 23 °C and humidity ranging from 45 to 55%, under a 12 h dark/light cycle. All the procedures during animal experiments were approved by the Institutional Animal Care and Use Committee at First Hospital of Jilin University (Approval No. 20220742). Melanoma skin cancer model was constructed by inoculation of B16 cells at a density of 5 × 10^5^/200 μL to the dorsal flank of C57BL/6 mice. After 14 d, the average size of the tumor reached 5–6 mm. Then, PBS, Ce6, Fe-CDs, and Fe-CDs@Ce6 conjugate (2 mg/20 g of mice body weight per day) were injected into tail vein, respectively. Then, a portable 660 nm laser with a power density of 100 mW/cm^2^ was applied to irradiate the tumor area of mice injected with Ce6 and Fe-CDs@Ce6, and maintained at the same distance (~ 10 cm) for 10 min. The tumor-bearing mice were sacrificed on 21 d, and then the tumor tissues were excised, weighed and photographed.

### In vivo imaging and ex vivo metabolic imaging experiment

For in vivo drug tracking, near-infrared dye IRDye^®^ 800CW NHS ester was purchased from LI-COR Biosciences (Lincoln, NE), and coupled with Fe-CDs to achieve in vivo imaging capacity. Briefly, the solution of Fe-CDs (20 mg/mL) was mixed with the solution of IR800 (1 mg/mL) in equal volumes and subjected to ultrasonic treatment at room temperature for 12 h. After that, the solution was dialyzed for 24 h with deionized water, and the powder was obtained through freeze-drying for subsequent applications. Each tumor-bearing mouse received an intravenous injection of 20 mg of Fe-CDs conjugated with IR800 through the tail vein. The experimental animals were anesthetized with isoflurane 1 h after injection, and then in vivo fluorescence imaging was performed using the Odyssey infrared imaging system (Odyssey, LI-COR, USA). For in vitro imaging of important organ metabolism, the animal models, injection methods, and observation time points were consistent with in vivo imaging, including 30 min, 1, 24, 36, and 48 h. The mice were pre-anesthetized with isoflurane, then the tissue including heart, liver, spleen, lung, kidney, and tumor were isolated. After collecting the tissues, they were grouped and photographed with in vivo fluorescence imaging system.

### Magnetic resonance imaging (MRI) experiment

Magnetic resonance imaging (MRI) experiments were conducted using a 7 T whole mouse MRI system (Varian 7T/210/ASR, Varian/Agilent) equipped with a 38 mm mouse body coil. To assess the *T*_*2*_ relaxation time, a Multi-Echo Multi-Slice (MEMS) sequence was employed, utilizing the following parameters: a repetition time (TR) of 1500 ms, 28 echoes, echo time (TE) ranging from 9 to 252 ms with 9 ms increments, data matrix of 128 × 128, and a Field of View (FOV) of 50 × 50 mm. *T*_*2*_-weighted images were acquired using a Fast Spin-Echo Multi-Slice (FSEMS) sequence with the following settings: TR = 2000 ms, TE = 24 ms, echo train length (ETL) = 4, k-zero = 2, matrix size of 256 × 256, FOV of 50 × 50 mm^2^, 20 interleaved slices with no gap and a slice thickness of 0.5 mm, and an average of 2 scans. Subsequently, the images were converted into DICOM format for viewing and analysis.

### Histological analysis

To evaluate the anti-cancer efficacy of Fe-CDs, the tumor tissues were harvested for the histological analysis, with PBS used as a control. After tail vein injection of mice with Ce6, Fe-CDs or Fe-CDs@Ce6, the whole tumor tissues were dissected and then fixed in 4% paraformaldehyde solution. The samples were encased in paraffin and sliced into sections measuring 3 μm. Then, hematoxylin and eosin (H&E) immunohistochemical stain was performed and analyzed with microscopic examination.

The TUNEL assay was performed using the TUNEL detection kit (C1086, Beyotime, China) following the provided instructions. After washing twice with PBS, the samples underwent treatment with 0.3% Triton X-100 (P0097, Beyotime, China) for 15 min. Subsequently, 50 µL of the TUNEL reaction mixture (5 µL TdT enzyme solution and 45 µL labeling solution) was applied, and the resulting mixture was incubated for 1 h at 37 ℃ in dark and humidified environment. The cells were co-stained with DAPI and visualized using laser confocal microscope.

Immunohistochemistry (IHC) was utilized for tumor tissue staining, employing primary antibodies (Abcam, U.K.) as follows: β-catenin (1:1000, ab32572), LEF1 (1:500, ab137872), HO1 (1:20,000, ab189491), and GPX4 (1:100, ab125066). Subsequently, the samples underwent staining with isotype-matched secondary antibodies for 2 h at room temperature. The secondary antibodies used were from the specific IHC polymer detection kit HRP/DAB (1:1000, ab209101, Abcam, U.K.). The stained tissues were then observed under a bright-field microscope.

### mRNA transcriptome sequencing (RNA-seq) and proteomic analysis

To verify the anti-cancer efficacy of Fe-CDs, high-throughput genome-wide RNA-seq was performed. After tail vein injection of mice with PBS, Fe-CDs, or Fe-CDs@Ce6, a portable 660 nm laser with a power density of 100 mW/cm^2^ was irradiated onto the tumors of Fe-CDs@Ce6 mice for 10 min at the same distance (~ 10 cm) to maintain a consistent laser power. The treatment continued for 7 d, after which the entire tumor tissues were dissected and collected. The tissue samples were immediately placed into liquid nitrogen and stored at − 80 °C. RNA integrity was evaluated using the RNA Nano 6000 Assay Kit on the Bioanalyzer 2100 system (Agilent Technologies, CA, USA). Total RNA served as the input material for RNA sample preparation. The obtained data were subjected to strict quality control, mapping to the reference genome, and prediction of new transcripts. Gene expression levels were quantified and subjected to differential expression analysis. Gene Ontology (GO), Kyoto Encyclopedia of Genes and Genomes (KEGG) enrichment analysis, protein–protein interaction analysis, and weighted correlation network analysis of differentially expressed genes were further analyzed.

### General procedure for western blotting

Phosphatase and protease inhibitors (P002, NCM, China) were added to RIPA buffer for cell and tissue lysis, and total proteins were extracted from cells and tissues in the resulting supernatant. Protein concentrations in cell samples were determined using the BCA Protein Assay Reagent (P0010S, Beyotime, China). After lysis, proteins were separated by SDS-PAGE (150 V, 60 min) and subsequently transferred to a PVDF membrane (Bio-Rad) using a Trans-Blot wet transfer system (Bio-Rad, 400 mA, 25 min). The membranes were then incubated with the primary antibody in fresh blocking buffer at 4 °C overnight. The concentrations of the primary antibodies used were as follows: Anti-beta Catenin antibody (1:1000, ab32572, Abcam), Anti-Lef1 antibody (1:500, ab3137872, Abcam), Anti-xCT antibody (1:10,000, ab175186, Abcam), Anti-Glutathione Peroxidase 4 antibody (1:1000, ab125066, Abcam), and Anti-Heme Oxygenase 1 antibody (1:1000, ab68477, Abcam). Following the overnight incubation, the blots were washed with TBST (WB20500, NCM, China) and incubated with the dye-labeled IRDye 800CW Goat anti-Rabbit IgG Secondary Antibody (dilution of 1:15,000, catalog number: 926-32211, lot: D00825-14, LI-COR) in fresh blocking buffer at room temperature for 1 h. Subsequently, the blots were washed again in TBST. The molecular weight (MW) of each protein on the immunoblots was estimated using a Pre-Stained Standard (10–180 KD) (26616, Thermo, USA). Immunoblot images were quantified using Image J software. The relative levels of target proteins were normalized to β-Actin (dilution of 1:50,000, catalog number: AC026, clone: ARC51105-01, ABclonal).

### Gene expression by quantitative PCR

Total RNA was isolated from B16 cells using TRIzol reagent (T9424, Sigma-Aldrich, USA) following the manufacturer’s instructions. Gene expression levels were analyzed by real-time quantitative PCR (qRT-PCR). First-strand cDNA synthesis was performed using a cDNA synthesis kit (RR036A, TAKARA, Japan) with 2 μg of total RNA as the starting material and 2 μL of the prepared cDNA as a template. qRT-PCR was conducted using 2 × Real Star Fast SYBR qPCR Mix (SYBR Green with ROX; Enzymenomics, Genster, China) according to the manufacturer’s instructions. Mouse β-actin was used as an internal reference gene to determine relative gene expression levels. Gene-specific primers for real-time RT-PCR analysis were obtained from Shanghai Bioengineering Co., Ltd. The following primer pairs were used: Actin (forward primer sequences: 5′-CACTGCCGCATCCTCTTCCTC-3′, reverse primer sequences: 5′-CGCTCGTTGCCAATAGTGATGAC-3′); xCT (forward primer sequences: 5′-TCATCGGATCAGGCATCTTCATCTC-3′, reverse primer sequences: 5′-TACTCCACAGGCAGACCAGAAAAC-3′); GPX4 (forward primer sequences: 5′-GGATGAAAGTCCAGCCCAAGGG-3′, reverse primer sequences: 5′-CACGGCAGGTCCTTCTCTATCAC-3′); HO1 (forward primer sequences: 5′-CAGCCACACAGCACTATGTAAAGC-3′, reverse primer sequences: 5′-TGAGAGGTCACCCAGGTAGCG-3′); β-catenin (forward primer sequences: 5′-GGAACGCAGCAGCAGTTTGTG-3′, reverse primer sequences: 5′-CCGAGCAAGGATGTGGAGAGC-3′); Wnt3a (forward primer sequences: 5′-GTATGACAGTGCCTCGGAGATGG-3′, reverse primer sequences: 5′-AGGTTCGCAGAAGTTGGGTGAG-3′); LEF1 (forward primer sequences: 5′-ACATCAAATAAAGTGCCCGTGGTG-3′, reverse primer sequences: 5′-TCTGGACATGCCTTGCTTGGAG-3′).

### Statistical analysis

The data are expressed as means ± standard deviation from several separate experiments. Statistical analysis was conducted using Prism 8.4/8.5 software (GraphPad Software). Normal distribution was assessed using the Shapiro–Wilk test. If the data met the normality criteria, a one-way ANOVA with posthoc multiple comparison test was performed. If not met, the Kruskal–Wallis non-parametric test was employed. The p values for *p < 0.05, **p < 0.01, ***p < 0.001, ****p < 0.0001 were considered statistically significant, NS indicates that there is no statistically significant difference.

## Results and discussion

### Synthesis and characterization of Fe-CDs@Ce6

**Fig. 1:**
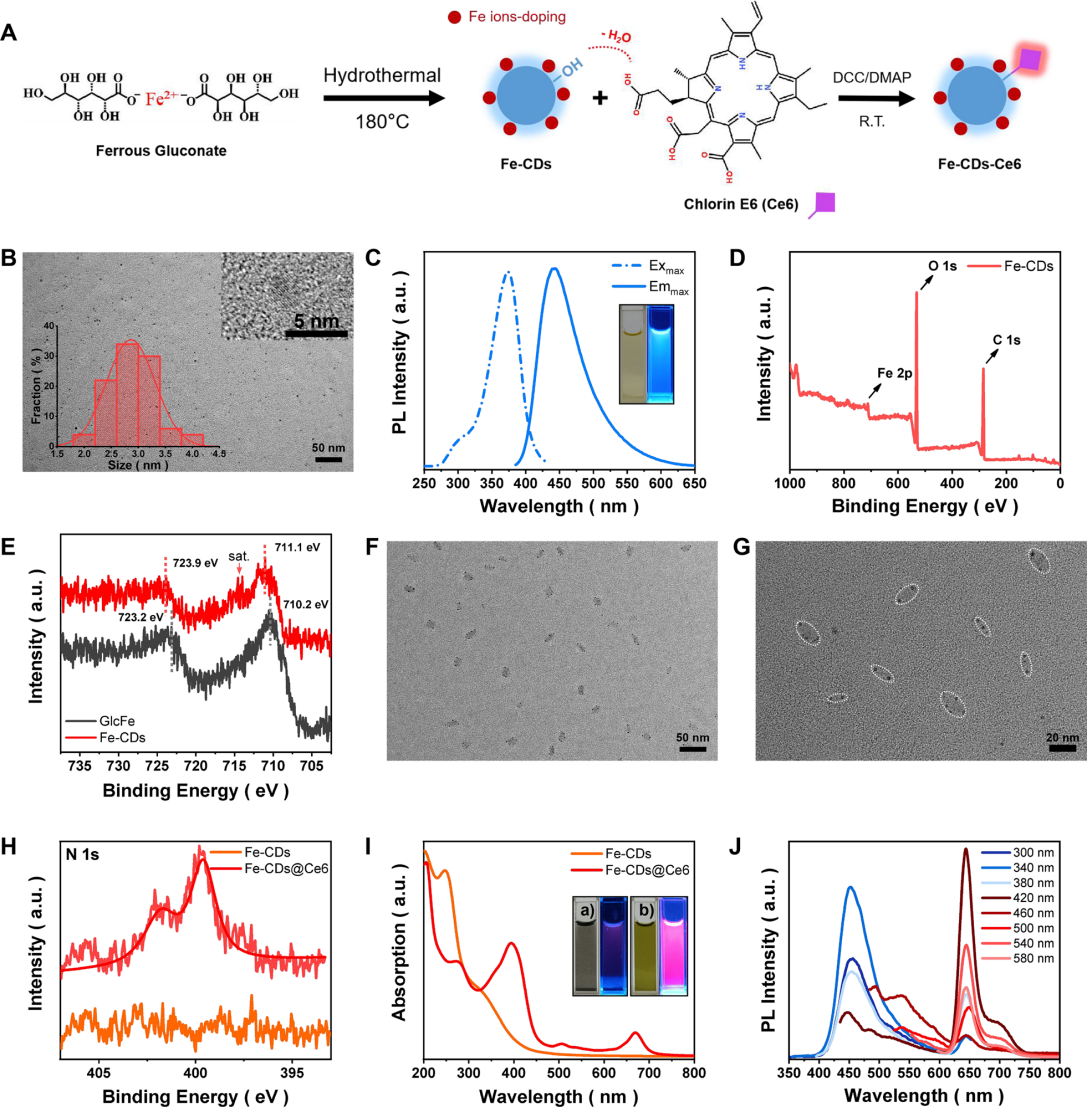
Characterization of Fe-CDs and Fe-CDs@Ce6. **A** Schematic illustration of the synthetic route of Fe-CDs and Fe-CDs-Ce6. **B** TEM image of Fe-CDs (inset: HR-TEM image and histogram of size distribution of Fe-CDs). **C** Maximum excitation and emission PL spectra of Fe-CDs (inset: photographs of Fe-CDs solution under daylight (left) and 365 nm UV light irradiation (right)). **D** XPS spectra of Fe-CDs. **E** Comparison of deconvoluted Fe 2p XPS spectra of GlcFe and Fe-CDs. **F-G** TEM images of Fe-CDs@Ce6 at low magnification and high magnification. **H** Comparison of deconvoluted N 1 s XPS spectra of Fe-CDs and Fe-CDs@Ce6. **I** UV–Vis absorption spectra of Fe-CDs and Fe-CDs@Ce6 (inset: photographs of (**a**) Ce6 and (**b**) Fe-CDs@Ce6 in PBS solution under daylight (left) and 365 nm UV light irradiation (right)). **J** PL spectra of Fe-CDs@Ce6 at different excitation wavelengths

In the present work, the multifunctional Fe-CDs@Ce6 was prepared by the coupling reaction between the hydroxyl groups on the surface of Fe-CDs and the carboxyl groups of the PDT sensitizer Ce6 (Fig. 1A). Firstly, the blue fluorescent Fe-CDs were synthesized by the hydrothermal reaction of ferrous gluconate, an FDA-approved iron supplement, on the basis of our previous work [45]. It was considered that the polysaccharide portion of gluconate salts would undergo dehydration and carbonization during the hydrothermal procedure, subsequently forming the carbon core of Fe-CDs. The Fe ions were in-situ doped within Fe-CDs, which could be connected to the hydroxyl and carboxylate groups via electrostatic interaction or might accumulate inside the graphite core. As shown in Fig. 1B, Fe-CDs appeared as a uniform distribution of nanoparticle morphology, with an average diameter of 2.8 nm. The high-resolution TEM displayed the lattice fringe structure in a single Fe-CDs, and the 0.21 nm lattice spacing corresponded to the (100) crystal plane of graphite carbon [46], confirming the carbon core structure. The yellowish solution of Fe-CDs exhibited typical blue fluorescence under UV irradiation (Fig. 1C). The PL spectra showed that the maximum excitation and emission wavelengths of Fe-CDs were 374 and 442 nm, respectively. Under the excitation of different light wavelengths, Fe-CDs presented obvious excitation-dependent behavior, similar to most of the reported CDs (Additional file 1: Fig. S1).

The XPS analyze verified the existence of C, O and Fe elements within Fe-CDs (Fig. 1D), and the atomic percentage was calculated as 70.2% for C, 24.6% for O, and 5.2% for Fe. The element composition with C as the main component and the low content of iron ensured the superior biocompatibility of Fe-CDs. The deconvoluted C 1 s spectra revealed four types of carbon, i.e., sp^2^ C (C=C), sp^3^ C (C–H/C–C), C–O, and C=O at the binding energy of 284.5, 284.9, 286.3, and 288.5 eV, respectively (Additional file 1: Fig. S2A). Besides, two oxygen bonding forms of C–O and C=O at 532.4 and 531.5 eV appeared in the O 1 s spectra of Fe-CDs (Additional file 1: Fig. S2B), indicating the constitute of carbonaceous skeleton and surface oxygen-containing functional groups in Fe-CDs. Noteworthy, the Fe 2p spectra of Fe-CDs showed double peaks at 711.1 (Fe 2p_3/2_) and 723.9 eV (Fe 2p_1/2_), which were 0.9 and 0.7 eV higher than those of the precursor GlcFe, respectively (Fig. 1E), indicating the oxidized valence state of Fe ions [47]. In addition, the Fe 2p_3/2_ peak of Fe-CDs was accompanied by an adjacent characteristic satellite peak, which was ascribed as the oxidized ionic form of Fe [48]. This was possibly due to the oxidation of Fe ions in the raw precursor caused by the high temperature and oxygen ambience from hydrothermal conditions. It was precisely because the Fe ions in Fe-CDs were in oxidized states that they could be selectively reduced to Fe^2+^ in a tumor environment with high GSH levels and triggered Fenton reaction, without causing fluctuations in oxidative stress and side effects of normal cells.

Then, Ce6 was anchored to the surface of Fe-CDs through DCC/DMAP-catalyzed condensation reaction. On one hand, the ferroptosis effect of Fe-CDs and the PDT function of Ce6 could synergistically increase the oxidative stress level and improve the tumor killing effect. On the other, as an aqueous-dispersed nanocarrier with high cell uptake efficiency, Fe-CDs could solve the problem of poor solubility of Ce6, which was conducive to its direct entry into tumor cells to exert phototherapy role. The obtained Fe-CDs@Ce6 presented a capsule-like assembly consisting of 2–4 nanoparticles (Fig. 1F–G). We speculated that this was because of the abundant hydroxyl groups on the surface of Fe-CDs and the three carboxyl groups of Ce6, thus the coupling reaction between Fe-CDs and Ce6 was not a one-to-one correspondence. Due to the presence of steric hindrance, the equilibrium consequence was that several Fe-CDs and Ce6 assembled together, forming a nanocapsule with the longitudinal length of ca. 20 nm. The increased size and the spindle structure might further facilitate the efficient cellular entry of Fe-CDs@Ce6 by endocytosis pathway. Besides, Fourier Transform-infrared spectroscopy (FT-IR) was conducted to further prove the successful connection of Fe-CDs and Ce6 (Additional file 1: Fig. S3). The stretching vibrations of O–H, –CH_2_–, C–O, and C–O–C at the wavenumber of 3362, 2925, 1394, and 1084 cm^−1^ were observed in both Fe-CDs and Fe-CDs@Ce6, similar to the bonding types in XPS results (Additional file 1: Fig. S4). However, a stretching vibration peak of ester bond (CO-OR) at 1780 cm^−1^ appeared in Fe-CDs@Ce6, which was originated from the condensation between Fe-CDs and Ce6. Also, the peak position of C=O at 1634 cm^−1^ shifted, probably indicating that the chemical environment of carbonyl groups changed after the coupling reaction of Ce6. Moreover, the N 1 s signal that only presented in the XPS elemental analysis of Fe-CDs@Ce6 was derived from the four pyrrole N rings in the molecular structure of Ce6 (Fig. 1H), which directly determined the modification of Ce6. The two new characteristic peaks at 670 and 506 nm in the UV–Vis absorption spectra of Fe-CDs@Ce6 also resulted from Ce6 (Fig. 1I). It was worth mentioning that Ce6 aggregated and precipitated in PBS because of its poor water solubility, leading to the quenching of fluorescence, while the solution of Fe-CDs@Ce6 exhibited a clear and transparent state with bright red fluorescence, which demonstrated that Fe-CDs could serve as effective nanocarriers of Ce6. As shown in Fig. 1J, the as-prepared Fe-CDs@Ce6 possessed two emission peaks constituted of blue fluorescence from Fe-CDs and red fluorescence from Ce6. Also, it could be seen that Fe-CDs@Ce6 could exist stably for a long time in the dispersants required for biological experiments, such as phosphate buffered salt solution (PBS), acidic PBS and serum, without aggregation or precipitation (Additional file 1: Fig. S5). The above results concluded that a type of Ce6-modified Fe ions-containing nanocapsule was successfully synthesized, which could be applied for activating ferroptosis and PDT synergistic therapy in tumor cells.

### Cytotoxicity and photodynamic properties of Fe-CDs@Ce6

**Fig. 2:**
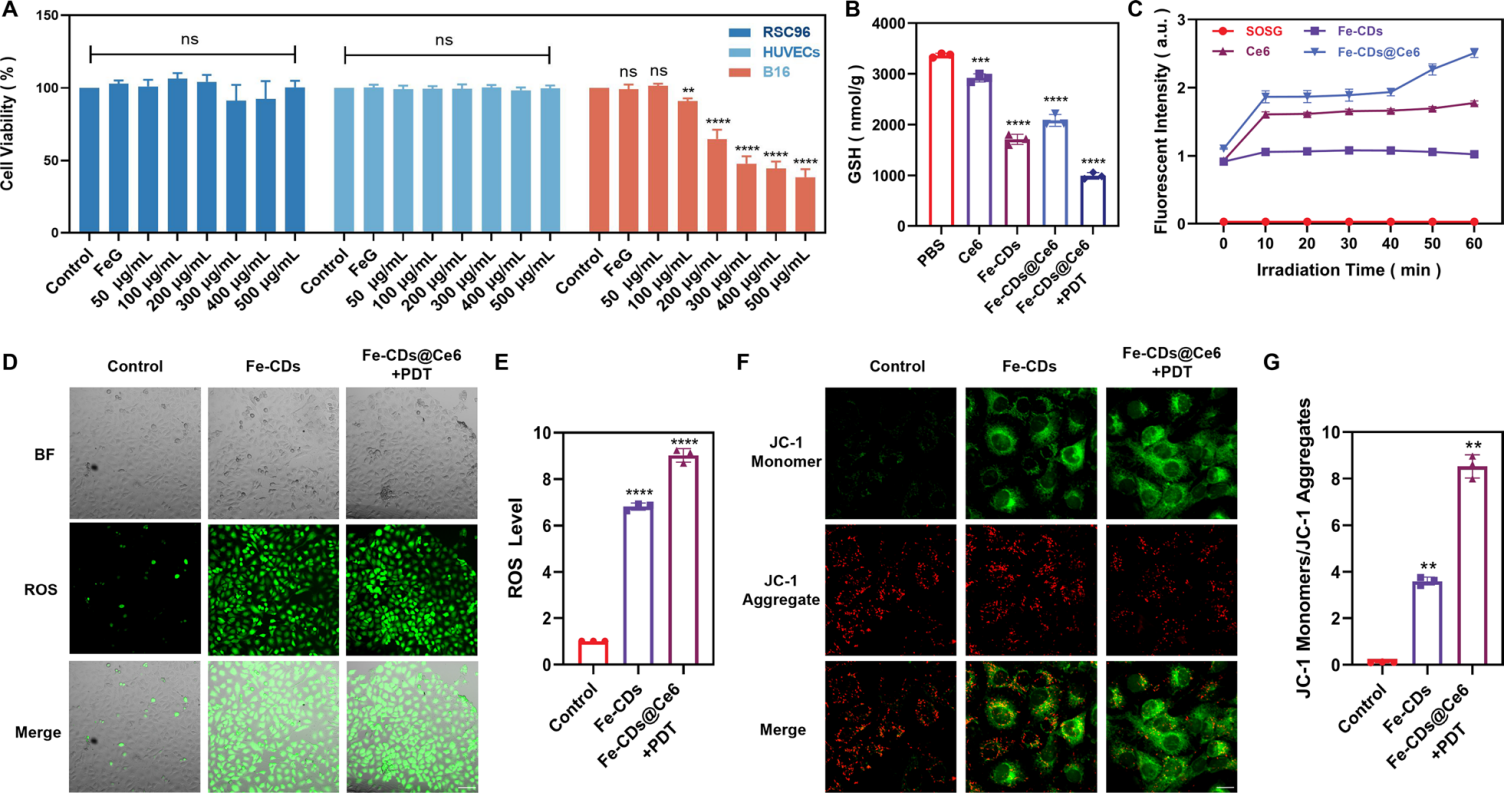
In vitro assay of cytotoxicity and oxidative stress. **A** Cell viability was assessed by CCK-8 assay. **B** GSH content detection.** C** The detection of singlet oxygen in the solution. **D** Detection of reactive oxygen species (ROS) levels in human umbilical vein endothelial cells co-cultured with B16 cell culture medium. Scale bar: 500 μm. **E** ROS levels. **F** Detecting changes in mitochondrial membrane potential in human umbilical vein endothelial cells co-cultured with B16 cell culture medium. Scale bar: 100 μm. **G** Quantification of the ratio of red signal (JC1 aggregates) to green signal (JC1 monomers), normalised to the intensity of the signal in non-treated control cells. (n = 3, **p < 0.01, ***p < 0.001, ****p < 0.0001 were considered statistically significant)

As shown in Fig. 2A, rat Schwann cells (RSC96), human umbilical vein endothelial cells (HUVECs) and mouse melanoma cells (B16) were selected for cytotoxicity assay of Fe-CDs. Compared with the control group, there were no significant differences in cell activities of RSC96 and HUVECs treated with Fe-CDs, even at a high concentration of 500 μg/mL. However, the cell viability of B16 cells decreased by about 20% upon the addition of 100 μg/mL Fe-CDs. When the concentration raised to 200 μg/mL, the survival rate of B16 cells was only 64%, and as the concentration of Fe-CDs continued to increase to 500 μg/mL, only 38% of B16 cells survived, indicating the efficient ability of Fe-CDs to selectively kill melanoma cells. The main mechanism inducing ferroptosis in tumor cells is lipid peroxidation and apoptosis caused by oxidative stress (OS), which mainly depends on the production of singlet oxygen (^1^O_2_), hydroxyl radicals (•OH) and peroxides. Nevertheless, cancer cells have developed a robust antioxidant defense system [49, 50], with glutathione (GSH) serving as a distinctive antioxidant to counteract oxidative stress [51]. It was speculated that the oxidized Fe ions in Fe-CDs would be reduced to Fe^2+^ under the high GSH level of tumor cells, triggering Fenton-like reactions, and resulting in selective killing of tumor cells. To prove this statement, the valence change of Fe ions in Fe-CDs after GSH treatment was explored by the chromogenic reaction with salicylic acid (SA) (Additional file 1: Fig. S6). It could be observed that the SA solution appeared a purple-red complex color with Fe^3+^ from Fe-CDs, while there was no change in the presence of FeSO_4_. As expected, Fe-CDs did not induce the coloration of SA after treated with GSH, suggesting the GSH-responsive reduction process of Fe ions in Fe-CDs. Noteworthy, not all Fe^2+^ is oxidized to Fe^3+^ during the preparation of Fe-CDs. There still remains some Fe^2+^ in Fe-CDs, as proved by the chromogenic reaction results of Fe^2+^ and potassium ferricyanide (K_3_[Fe(CN)_6_]) (Additional file 1: Fig. S7). Moreover, in the presence of H_2_O_2_ and GSH-treated Fe-CDs, the methylene blue (MB) indicator was degraded (Additional file 1: Fig. S8), which could be attributed to the Fenton-like reaction induced by GSH-stimulated Fe^2+^ release from Fe-CDs [52]. Since most of Fe ions have been oxidized to Fe^3+^, the catalytic effect of Fe-CDs is more significant after GSH reduction, which is conducive to the selective killing of tumors with high GSH expression milieu. In addition, the measurement of glutathione (GSH) levels was also conducted by detection of oxidized glutathione (GSSG) in Fe-CDs and Fe-CDs@Ce6 treated B16 cell. As illustrated in Fig. 2B, the cellular GSH content decreased by 49.7% after treatment with Fe-CDs compared to the control group. Upon combined 660 nm laser irradiation, the GSH level further decreased to 29.22% compared to the control group, which indicated that Fe-CDs could consume GSH in tumor cells, and the depletion of GSH becomes more pronounced when introduced PDT. The endoperoxide derived from the interaction of SOSG with ^1^O_2_ exhibited green fluorescence, thus the light intensity was recorded to confirm whether Fe-CDs@Ce6 could generate a high concentration of ^1^O_2_ under laser irradiation that subsequently promoted ferroptosis and affected the tumor microenvironment. For SOSG and Fe-CDs, there were no changes in the fluorescent intensity with the extension of laser irradiation time, while Ce6 and Fe-CDs@Ce6 both produced high levels of singlet oxygen under irradiation (Fig. 2C). Moreover, the fluorescent intensity of Fe-CDs@Ce6 was significantly stronger than that of Ce6 alone, which was attributed to the improved solubility of Ce6 in PBS by Fe-CDs as nanocarriers.

The consumption of GSH and the oxidative damage induced by ferroptosis can enlarge the therapeutic function of PDT, also integrating PDT will in turn accelerate the occurrence of ferroptosis [53]. Therefore, in order to investigate the synergistic effect between Fe-CDs and Ce6, the levels of ROS and changes in mitochondrial membrane potential in cells were measured by fluorescence staining. The B16 cells were first treated with Fe-CDs and Fe-CDs@Ce6 combined with PDT, and then the conditioned medium was isolated and co-cultured with HUVECs, which was incubated to simulate vascular cells within tumor microenvironment (TME). The results showed that HUVECs cultured in Fe-CDs-treated B16 medium had a 6.8-fold higher ROS production compared to the control group, indicating the ability of Fe-CDs to induce oxidative damage of vascular cells in TME. Furthermore, the B16 medium treated with Fe-CDs@Ce6 and PDT induced higher ROS levels in HUVECs, which was 9.0 fold that of the control group (Fig. 2D–E). It was speculated that the possible mechanism was that Fe-CDs@Ce6 + PDT treatment significantly promoted the death of tumor cells and caused the release of inflammatory factors. Then, when HUVECs were cultured in the B16 cell conditioned medium, inflammation-related response pathways were activated, thereby promoting ROS production in HUVECs. To verify this statement, the level of tumor necrosis factor (TNF-α) and interleukin (IL-10) were tested by enzyme linked immunosorbent assay (ELISA), which are increased and decreased in the conditioned medium after Fe-CDs@Ce6 + PDT treatment, respectively (Additional file 1: Fig. S9). Also, the mitochondrial membrane potential in HUVECs cultured with B16 conditioned medium after the treatments of Fe-CDs and Fe-CDs@Ce6 + PDT were 3.6 and 8.5 times higher than those of the control group, demonstrating the impaired mitochondrial function caused by ROS (Fig. 2F, G). Therefore, Fe-CDs might inhibit tumor blood supply by interfering with tumor vascularization, and this effect could be further enhanced under the intervention of PDT by accelerating the ROS production of Fe-CDs. In addition, Fe-CDs alone caused 5.9-fold ROS production and 5.0-fold mitochondrial membrane potential transition in B16 cells (Additional file 1: Fig. S10A–D), that is, Fe-CDs itself could efficiently kill melanoma cells through ROS, which was similar to the cell viability results. As a new type of Fe-doped nanomaterials, it is necessary to verify whether Fe-CDs cause ROS through ferroptosis pathway.

### Ability of Fe-CDs@Ce6 to inhibit melanoma cell proliferation by promoting ferroptosis

**Fig. 3:**
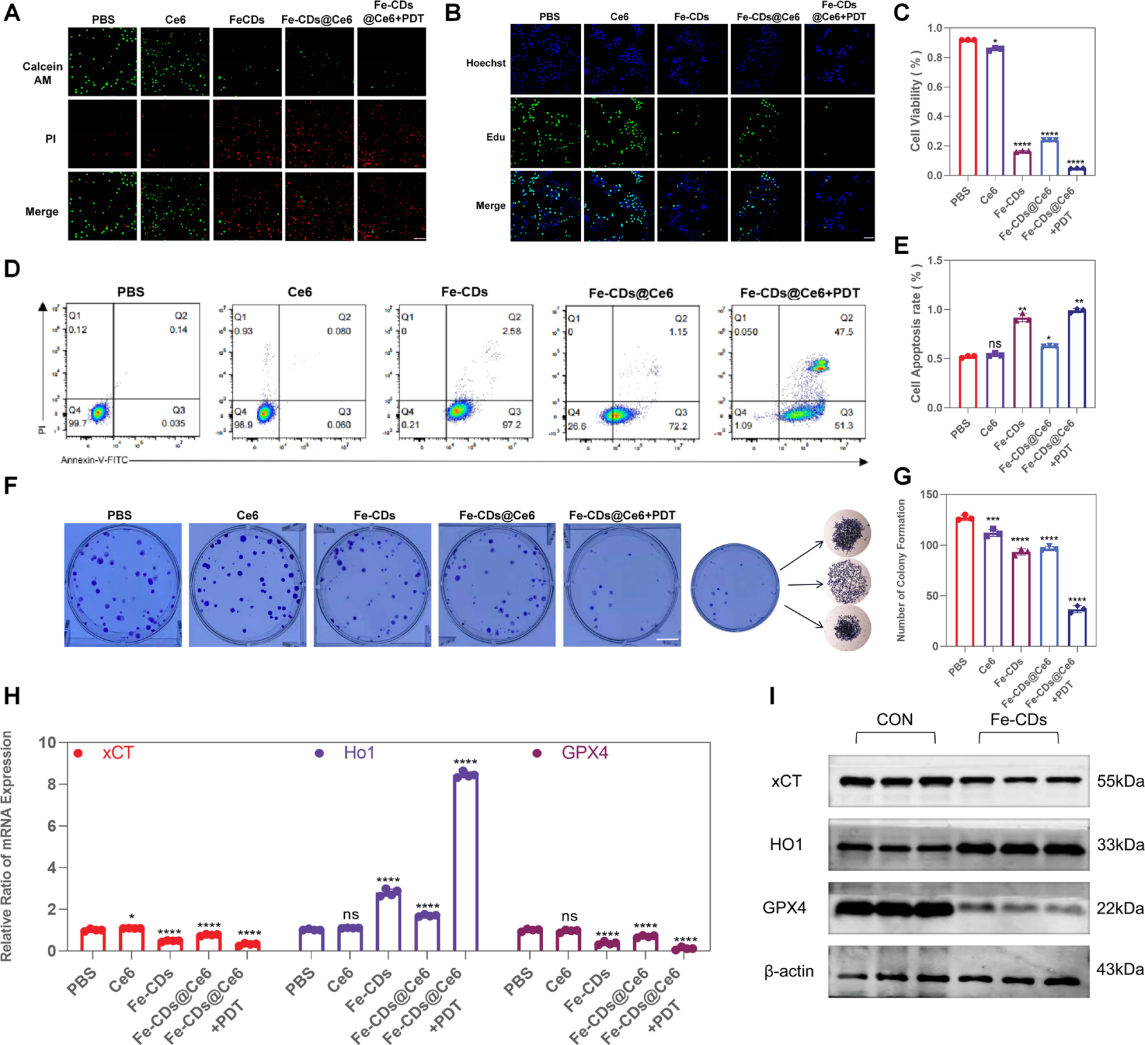
Evaluation of the ability to inhibit the proliferation of B16 cells. **A** B16 cells were treated with Fe-CDs and Fe-CDs@Ce6. Microscopy images were captured after staining with the LIVE/DEAD kit. Live cells are stained with green and dead cells with red (n = 3). Scale bar: 100 μm. **B** Cells were subjected to pulse-labeling with EdU as specified. Green fluorescence represents the EDU positive cells, and blue fluorescence represents the Hoechst stained cells. (n = 3). Scale bar: 600 μm. **C** Quantitative cell viability of the Live/Dead assay images. **D** Cell apoptosis of PBS, Ce6, Fe-CDs, Fe-CDs@Ce6, Fe-CDs@Ce6 + PDT by flow cytometry analysis. **E** Quantitative analyses of EdU assay. **F** Clone formation assay for assessing clone formation. Scale bar: 60 mm. **G** Histogram of cell clone formation rate. **H** The RNA expression levels of xCT, HO-1, and GPX4 (n = 4). **I** The protein expression levels of xCT, HO-1, and GPX4 (n = 3) (NS = not significant, *p < 0.05, **p < 0.01, ***p < 0.001, ****p < 0.0001)

Firstly, Calcein-AM/PI, EDU, Annexin V-FITC/PI and colony formation assays were performed to verify that Fe-CDs could inhibit the proliferation and induce apoptosis of B16 cells. The results of live/dead cell staining showed that about 91.9% and 85.7% of the cells showed green fluorescence in the control group and Ce6 group, respectively. In contrast, 83.6% of the B16 cells showed red fluorescence representing apoptosis in the Fe-CDs treatment group, and up to 95.1% cells underwent apoptosis in the Fe-CDs@Ce6 + PDT treatment group (Fig. 3A, C). Besides, the EDU staining results indicated that the inhibition rate of B16 cell proliferation was 91.6% after Fe-CDs treatment, while after modified with Ce6 and combined with PDT, approximately 100% of B16 cell proliferation were restrained (Fig. 3B, E). Moreover, the flow cytometry results of V-FITC/PI staining showed that 99.7% of the cells in the control group were located in the Q4 region of living cells. However, 97.2% of the cells in the Fe-CDs group were located in the Q3 region, especially there were 51.3% of the cells located in the Q3 region and 47.5% in the Q2 region for Fe-CDs@Ce6 + PDT group (Fig. 3D). The cell surface began to express phosphatidylserine and the cell membrane was relatively intact. Furthermore, the colony formation assay showed that 26.5% of the cell colony forming was inhibited in Fe-CDs group compared with the control group, and when combined with PDT the inhibiting effect could be as high as 71.1% (Fig. 3F, G), suggesting the strong ability of Fe-CDs against the proliferation of melanoma cells.

According to the recent studies, the activation of ferroptosis may help enhance the anti-tumor immune responses and prevent tumor progression, the inhibitory effect of Fe-CDs on the proliferation of B16 cells was likely to be related to ferroptosis. In general, ferroptosis is divided into two types, i.e., endogenous and exogenous pathways [54]. The exogenous pathway includes inhibition of cell membrane iron transporter proteins, such as the cystine/glutamate transporters (system Xc-), and activation of lactotransferrin induced ferroptosis. The endogenous pathway involves inducing ferroptosis by blocking the intracellular antioxidant enzyme GPX4. To further confirm whether the inhibition of B16 cell proliferation by Fe-CDs is related to ferroptosis, reverse transcription-polymerase chain reaction (RT-PCR) was conducted. The results showed that the expression levels of XCT (system Xc-) and GPX4 in B16 cells were reduced after treatment with Fe-CDs, while the expression level of Heme Oxygenase-1 (HO1) significantly increased, indicating the elevation of iron metabolism and up-regulation of ferroptosis (Fig. 3H). Furthermore, Western blotting (WB) experiments were performed on Fe-CDs treated B16 cells, which revealed a remarkable decrease in the protein expression levels of XCT/GPX4 and increase in that of HO1, consistent with the PCR results (Fig. 3I; Additional file 1: Fig. S11A–C). This further substantiated that Fe-CDs could induce ferroptosis, and the PDT treatment based on Fe-CDs@Ce6 greatly amplified the ferroptosis effect, thus achieving the function of inhibiting the proliferation of B16 cells.

### Ability of Fe-CDs@Ce6 to inhibit melanoma cell invasion by suppressing Wnt pathway

**Fig. 4:**
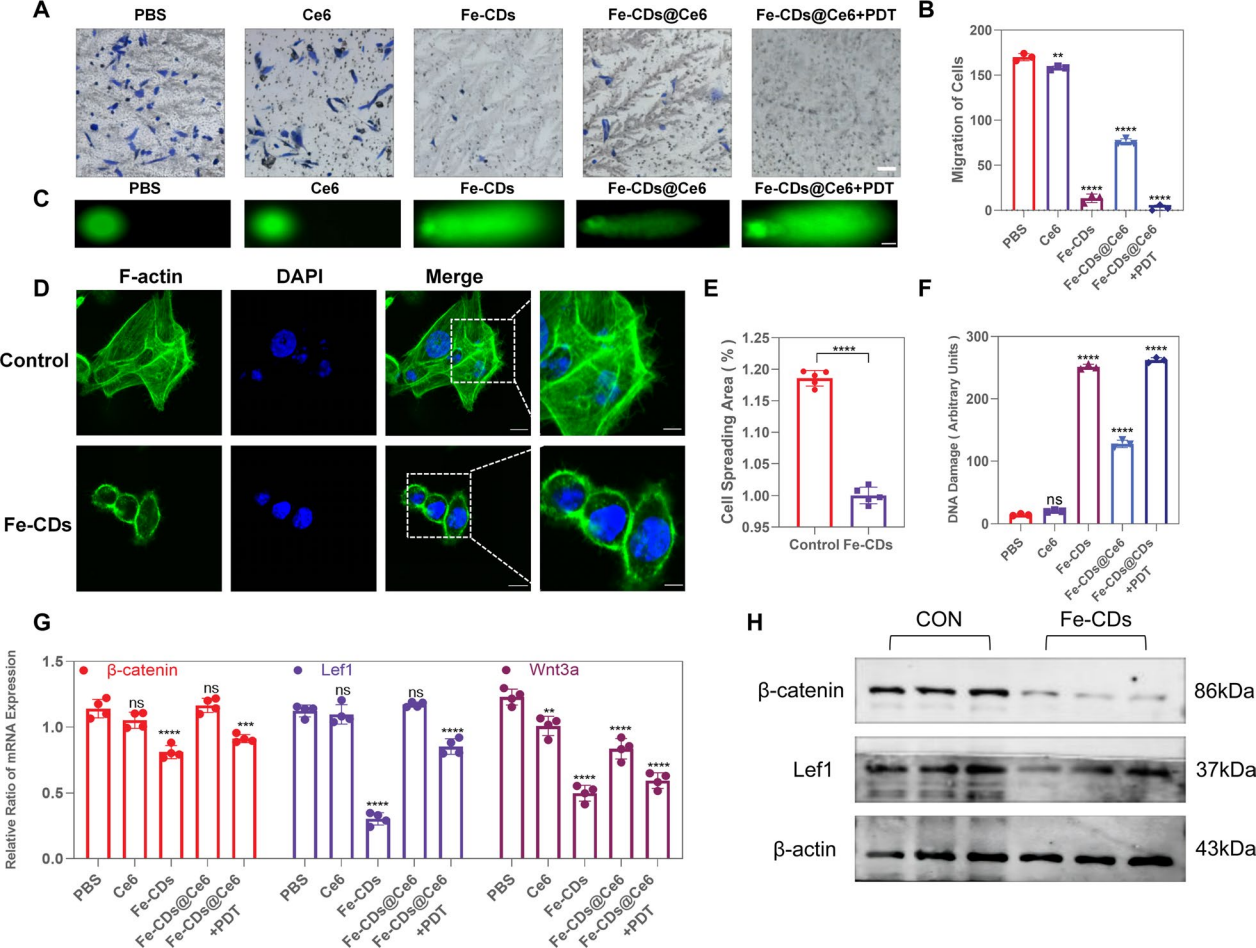
Evaluation of the ability to inhibit the proliferation of B16 cells. **A** The invasion of B16 cells was assessed using a transwell assay (n = 3). Scale bar: 50 μm. **B** Data statistics of cell invasion in transwell chamber. **C** The comet assay was employed to detect DNA fragmentation in B16 cells (n = 3). Scale bar: 50 μm. **D** Fe-CDs can reduce the F-actin level in B16 cells. The green fluorescence represents F-actin, and the blue fluorescence represents DAPI. Scale bar: 80 µm, 80 µm, 80 µm and 20 µm from left to right. (n = 5). **E** The length of the comet tail was measured as the distance from the leading edge of the nucleus to the tails end. **F** F-actin levels were quantified using ImageJ software. **G** The RNA expression levels of β-catenin, Lef1, and Wnt3a (n = 4). **H** Protein expression levels of β-catenin, Lef1, and Wnt3a (n = 3) (NS = not significant, *p < 0.05, **p < 0.01, ***p < 0.001, ****p < 0.0001)

Inhibition of tumor cell invasion can reduce the rate of tumor metastasis, because the active movement of tumor cells is an important initial migration process. Therefore, the effect of Fe-CDs on B16 cell invasion was evaluated by Transwell assay to explore whether Fe-CDs can also hinder tumor metastasis. As shown in Fig. 4A, it illustrated that the Fe-CDs, Fe-CDs@Ce6 and Fe-CDs@Ce6 + PDT groups had obviously reduced number of blue-stained chemotactic cells passing through Matrigel coating. Compared to the control group, the invasion rate of B16 cells in the Fe-CDs group was 7.8%, while after combined PDT, the invasion rate reduced to only 1.6% (Fig. 4B). Then, the DNA damage extent was evaluated by comet assay and calculated by quantifying the comet-tail moment of in Fig. 4C. It was shown that the treatment of Fe-CDs and Fe-CDs@Ce6 + PDT generated prominent DNA injury with 19.6 and 20.2 times than that of the control group (Fig. 4F). It was worth mentioning that the efficacy of Fe-CDs@Ce6 was not as good as that of Fe-CDs without laser irradiation. We believed the reason was to use the mass concentration of nanodrugs as a reference, and the amount of Fe-CDs contained in Fe-CDs@Ce6 group was lower compared to pure Fe-CDs group at the same mass concentration. However, with the introduction of PDT, the therapeutic impact of Fe-CDs@Ce6 was even better than that of Fe-CDs, which further demonstrated the synergistic amplification effect between ferroptosis and PDT. This was also conducive to reducing the dosage amount of drugs and precise tumor therapy under laser irradiation.

Moreover, in order to intuitively observe the degree of cell migrations, stress fiber polymerization assay was subsequently conducted on the Fe-CDs treated B16 cells using phalloidin to stain the cellular cytoskeleton. The cytoskeleton expansion of B16 cells treated with Fe-CDs appeared smoother compared to untreated cells, with a reduction of approximately 15.7% in cell spreading area (Fig. 4D, E), suggesting that Fe-CDs could influence the migration and invasion of B16 cells. In addition, the angiogenesis assay also showed that the tube formation ability of HUVECs cultured in the tumor cell medium after combined PDT treatment was significantly decreased (Additional file 1: Fig. S12). Based on above consequences, Fe-CDs could inhibit invasion and prevent metastasis of melanoma cells, as well as suppress angiogenesis within tumor environment. Previous studies had shown that the Wnt/β-catenin pathway could accelerate tumor cell infiltration and distant metastasis, thereby accelerating tumor progression. Therefore, we considered that Fe-CDs might inhibit B16 cell invasion and metastasis by influencing the Wnt/β-catenin pathway. Since β-catenin and lymphoid enhancer-binding factor 1 (Lef1) were two essential molecules in the Wnt/β-catenin pathway, RT-PCR experiments were then carried out to determine their expression levels. The results indicated a significant decrease in the expression of β-catenin, Lef1 and Wnt3a (Fig. 4G). Also, the results of WB experiments demonstrated the protein expressions of β-catenin and Lef1 were notably down-regulated (Fig. 4H; Additional file 1: Fig. S11D, E). In summary, the as-prepared Fe-CDs can inhibit the proliferation and migration of B16 cells by suppressing the Wnt pathway, which heralds the great potential of Fe-CDs serving as an anti-tumor functional nanocarrier of Ce6 and achieving ferroptosis/PDT synergistic treatment of melanoma in vivo.

### In vivo evaluation of the therapeutic effect of Fe-CDs@Ce6 on melanoma

**Fig. 5:**
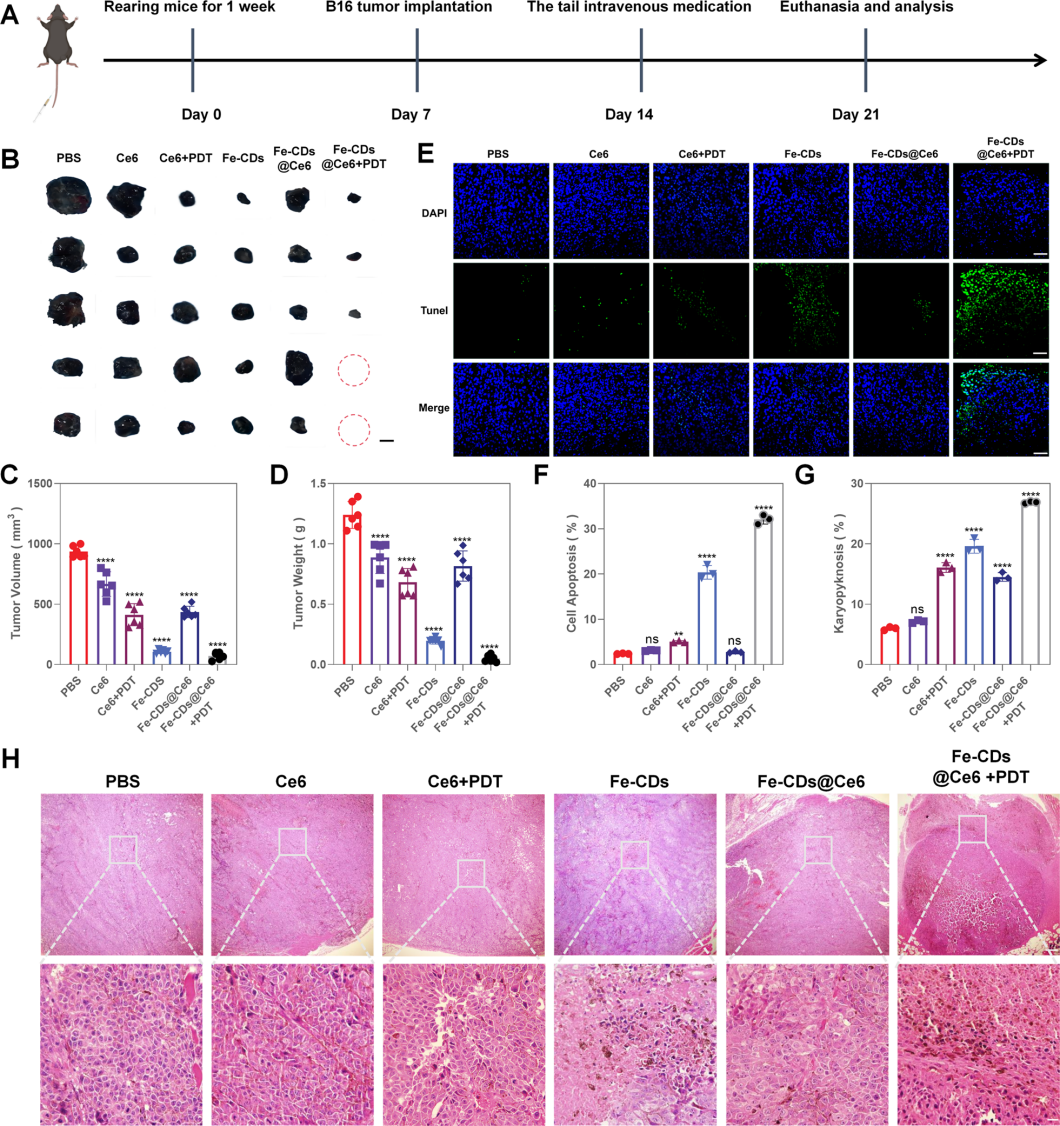
In vivo experiments of melanoma therapy. **A** The time schedule of this study. **B** Optical images of harvested tumors after the treatments (n = 6). Scale bar: 3 mm. **C** The volume of tumors from the mice in different groups (n = 6). **D** The weight of tumors from the mice in different groups (n = 6). **E** Tumor tissues were subjected to TUNEL staining following different treatments, with nuclei stained in blue and TUNEL-positive cells in green (n = 3). Scale bar: 100 μm. **F** Quantitative result of TUNEL assay was analyzed. **G** Quantitative result of hematoxylin–eosin staining was analyzed. **H** Staining of tumor tissues post-treatment using Hematoxylin and Eosin (H&E) (n = 3). Scale bar: 200 µm and 20 µm from top to bottom. (NS = not significant, *p < 0.05, **p < 0.01, ***p < 0.001, ****p < 0.0001)

After clarifying that Fe-CD@Ce6 possessed the function of inhibiting melanoma cell proliferation and vascularization in tumor condition, we then created a mouse melanoma model by subcutaneously injecting B16 cells into male BALB/c mice. When the average sizes of tumor volume reached 5–6 mm after a week, 36 tumor-bearing mice were randomly divided into 6 groups and continuously administered via tail vein injection with PBS, Ce6, Ce6 combined with PDT, Fe-CDs, Fe-CDs@Ce6 and Fe-CDs@Ce6 combined with PDT, respectively (Fig. 5A). After 7 days of treatment, all the mice were euthanized, whose tumors and organs were collected for further research. First, the tumor volume and mouse body weight were measured daily (Additional file 1: Fig. S13A), which revealed that the tumor volume continued to increase in PBS and Ce6 groups by about 20 times. However, the tumor volume growth rate of the Fe-CDs and Fe-CDs@Ce6 + PDT groups were significantly slower than those of the other three groups (Additional file 1: Fig. S13B). Remarkably, the tumors of two mice in the Fe-CDs@Ce6 + PDT group were even completely ablated, representing the efficient synergistic therapy effect (Fig. 5B). Noteworthy, due to the poor water solubility and uptake rate of Ce6, which leads to the fact that Ce6 cannot effectively reach the tumor site through intravenous administration, thus the killing effect is not ideal for Ce6 + PDT group. This further emphasizes the significance of Fe-CDs@Ce6 nanocomposite to improve the tumor enrichment of Ce6 for enhancing PDT effect. The statistical results of tumor volume and mass on 21 d also verified that the combined treatment of Fe-CDs@Ce6 + PDT had the best anti-tumor outcomes (Fig. 5C, D). The relatively poor efficacy of Fe-CDs@Ce6 group was due to the fact that the content of Fe-CDs in Fe-CDs@Ce6 was less than Fe-CDs under the same mass concentration, while introducing laser irradiation could achieve a better effect with reduced dosage. Besides, there were no significant changes in body weight among the five groups (Additional file 1: Fig. S13C), which suggested that the treatments did not cause malnutrition or consume normal organs, further confirming the good biocompatibility and target-free selective anti-tumor properties of Fe-CDs.

Then, TUNEL staining of apoptotic cells were performed in the tumor tissue samples obtained from the corresponding animal experiments. As shown in Fig. 5E, compared with other groups, the Fe-CDs and Fe-CDs@Ce6 + PDT groups showed obviously more green fluorescent staining cells than other groups, with apoptosis proportion of 20.4% and 32.0%, respectively (Fig. 5F). This further proved that Fe-CDs could promote the apoptosis of B16 cells and inhibit tumor growth, especially the effect was enhanced after combined PDT. Moreover, similar results were also presented in H&E staining images, which illustrated that the tumor tissues treated with Fe-CDs exhibited approximately 20% chromatin condensation, and about 27% chromatin condensation occurred after PDT treatment (Fig. 5G, H). However, there were no statistically significant differences in the PBS and Ce6 groups. Nuclear pyknosis is the irreversible condensation of chromatin in the nucleus, usually associated with necrosis or apoptosis. In vivo experimental results concluded that Fe-CDs@Ce6 could effectively inhibit melanoma growth by inducing tumor cell apoptosis and even eliminated tumors through combining with PDT.

### Tumor-targeting ability and in vivo biocompatibility of Fe-CDs@Ce6

**Fig. 6:**
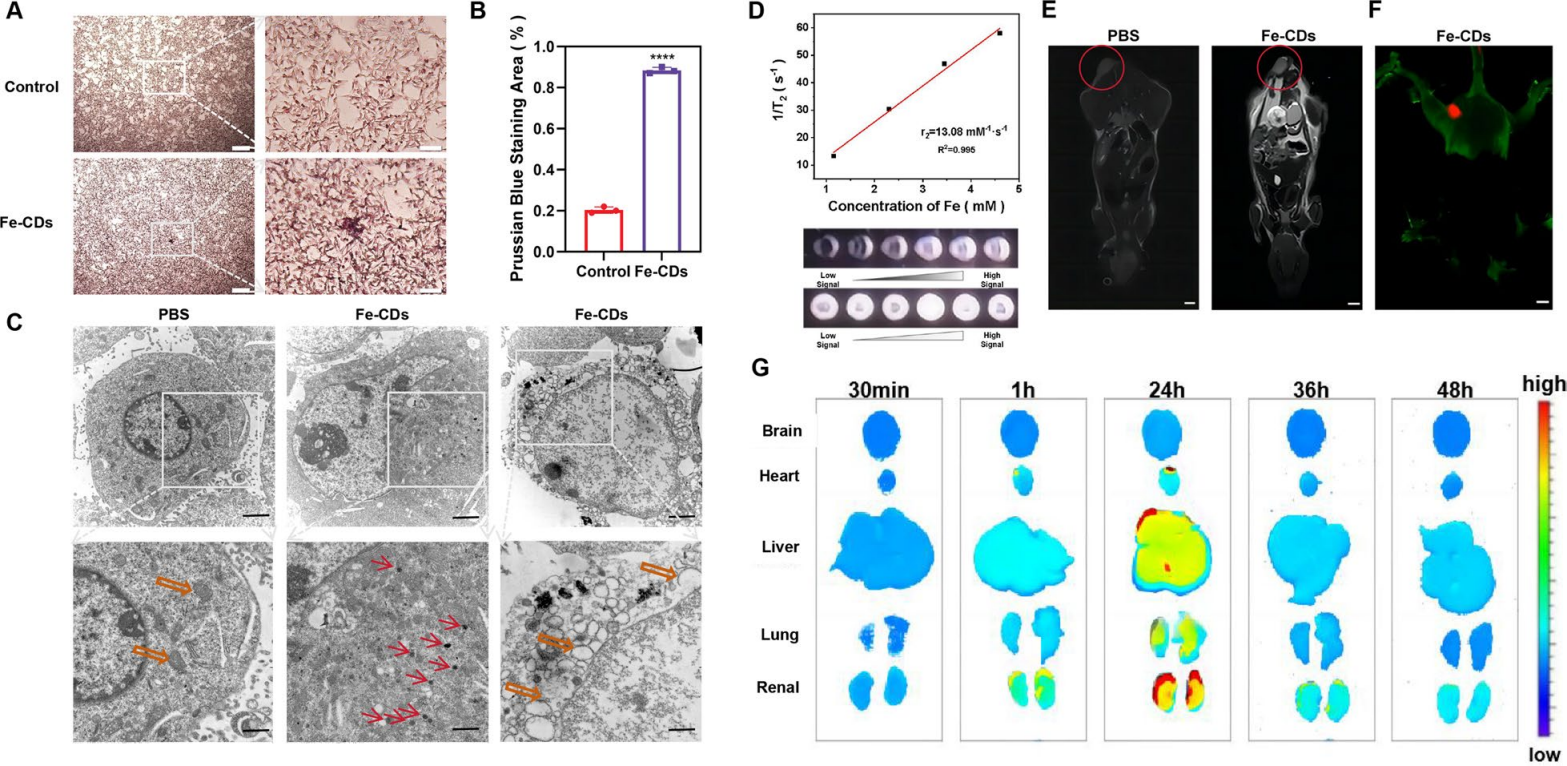
Tumor-targeting ability, magnetic resonance imaging (MRI) ability and in vivo metabolism assay. **A** The B16 cells treated with Fe-CDs, when fixed and stained with Prussian blue, exhibited a blue color indicative of iron (n = 3). Scale bar: 200 μm and 50 μm from left to right. **B** Quantitative result of Prussian blue was analyzed. **C** The B16 cells treated with Fe-CDs were fixed and photographed using transmission electron microscopy. The right figure shows mitochondrial vacuolization. Scale bar: 2 μm and 1 μm from top to bottom. **D** The linear relationship of Fe-CDs@Ce6 solutions at different concentrations, and *T*_*2*_-weighted MR images of Fe-CDs@Ce6 solutions at various concentrations. **E** In vivo *T*_*2*_-weighted MRI and the relative signal intensity of the tumor following administration of Fe-CDs@Ce6. Scale bar: 50 mm. **F** The tail vein injection of Fe-CDs@Ce6 combined with IR800 in mice was performed to observe representative images of the distribution of Fe-CDs@Ce6 in the mouse body. Scale bar: 50 mm. **G** Representative fluorescence images of dissected organs at 30 min, 1, 24, 36, and 48 h after the injection of Fe-CDs@Ce6-IR800. From top to bottom: brain, heart, liver, lungs, and kidneys (n = 3, ****p < 0.0001)

As mentioned above, Fe-CDs@Ce6 can exert anti-tumor effect without affecting the survival status and body weight of mice. This may be due to the enhanced permeability and retention (EPR) effect of Fe-CDs@Ce6 nanostructures or the increased permeability of tumor blood vessels leading to the local uptake of Fe-CDs@Ce6 [55, 56]. To confirm whether Fe-CDs could enter B16 cells, Prussian blue staining of Fe ions was used to label intracellular Fe-CDs. It could be observed that 88% of the cells in the Fe-CDs group exhibited blue-stained cytoplasm, whereas only 20% cells in the control group showed blue cytoplasm (Fig. 6A, B), proving the high cell entry efficiency of Fe-CDs. According to the previous reports, CDs can enter cells through clathrin or caveolin-mediated endocytosis due to their nano-size effect and good biocompatibility, which make CDs an ideal drug carrier. In order to determine whether Fe-CDs could carry Ce6 into cells efficiently, confocal microscopy was used to record the uptake of Fe-CDs@Ce6 based on the intrinsic red fluorescence of Ce6. The obvious fluorescence signal could be seen within the cytoplasm of B16 cells, and the fluorescence intensity at the incubation temperature of 37 °C was higher than that at 4 °C (Additional file 1: Fig. S14). The decrease of enzyme activity under low temperature conditions would interfere with the energy production of mitochondria, indicating that the uptake of Fe-CDs@Ce6 might be energy-dependent endocytosis. Also, the efflux pathway of Fe-CDs@Ce6 was monitored, which could be significantly inhibited at 4 °C, further indicating that the exclusion process of Fe-CDs@Ce6 was also energy-dependent (Additional file 1: Fig. S15). Moreover, transmission electron microscopy (TEM) identified the spherically shaped black nanoparticles within the cytoplasm of B16 cells (Fig. 6C). In addition, Fe-CDs caused vacuolar changes in the mitochondria of B16 cells, indicating the mitochondrial oxidative damage and apoptosis in B16 cells.

Benefiting from the doped Fe ions in Fe-CDs, it is expected to be used as a magnetic resonance imaging (MRI) contrast nanoagent to achieve drug tracking and tumor diagnosis. Therefore, the *T*_*2*_ relaxation rate (*r*_*2*_) of Fe-CDs was measured and fitted as 13.08 mM^−1^ s^−1^ according to the transverse relaxation times of Fe-CDs at different concentrations (Fig. 6D), which were comparable to the conventionally used *T*_*2*_-weighted MRI contrast agents (Fe_3_O_4_ nanoparticles) [57]. The MRI photographs of Fe-CDs solutions at different concentrations also verified the excellent *T*_*2*_-weighted MRI capability of Fe-CDs, exhibiting the potential of MRI-guided tumor therapy. Based on the above results, a marked white MRI signal was clearly observed in the melanoma area of mice after tail vein injection of Fe-CDs for 30 min, suggesting that Fe-CDs could be accurately accumulated at the tumor sites (Fig. 6E). Furthermore, upon intravenous injection of Fe-CDs conjugated with near-infrared dyes (IR800) in mice for 30 min, a significant accumulation of Fe-CDs was observed at the tumor site (Fig. 6F), corresponding to the MRI results. In addition, fluorescence quantification was performed by dissecting the major organs of the mice at 30 min, 1, 24, 36 and 48 h after Fe-CDs injection (Fig. 6G). It was found that Fe-CDs were mainly metabolized from the body via liver and kidneys, which was similar to the most reported CDs. After 48 h of injection, over 90% of Fe-CDs was metabolized and no significant fluorescence signal was detected, indicating the fast plasma clearance rate of Fe-CDs. Finally, H&E staining of important organs including heart, liver, spleen, lung and kidney showed that continuous administration of Fe-CDs and Fe-CDs@Ce6 did not cause obvious inflammatory infiltration or cell state changes, confirming the satisfactory biosafety of Fe-CDs (Additional file 1: Fig. S16). In summary, a biocompatible and simple ferroptosis/PDT/MRI multi-functional nanoplatform was constructed based on Fe-CDs@Ce6, showing broad application potential in the treatment of melanoma and other types of tumors.

### mRNA transcriptome sequencing (RNA-seq) and bioinformatics analysis

**Fig. 7:**
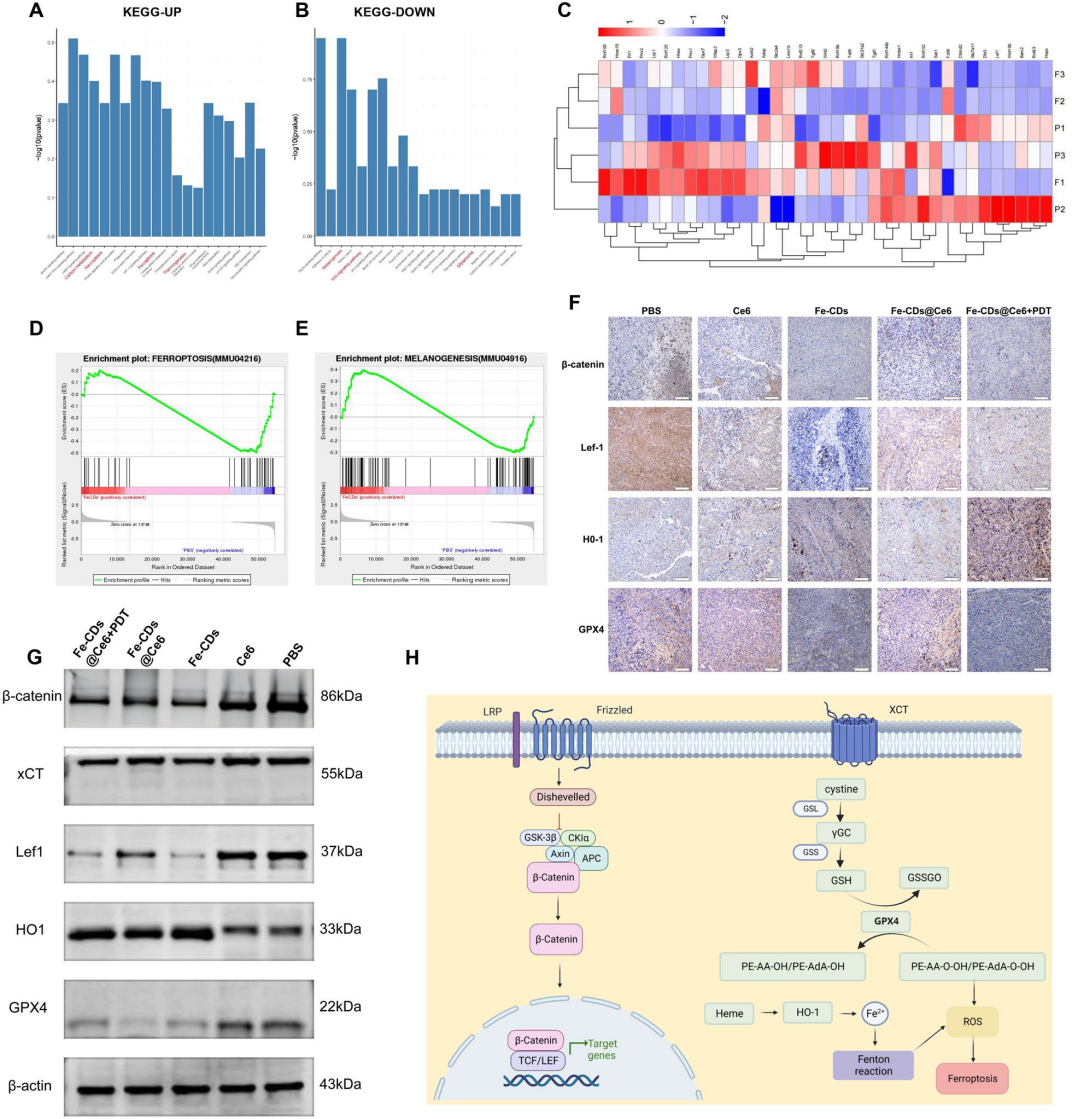
Analysis of mRNA expressions through transcriptome sequencing after combined therapeutic intervention. **A** The KEGG analysis revealed 20 pathways significantly enriched with upregulated differentially expressed genes. **B** The KEGG analysis revealed 20 pathways significantly enriched with downregulated differentially expressed genes. **C** Heat map diagram based on differentially expressed genes from the control and test groups. **D** GSEA enrichment plots of differentially expressed genes centralized in ferroptosis signaling pathway. **E** GSEA enrichment plots of differentially expressed genes centralized in melanogenesis signaling pathway. **F** The tissue sections obtained from treated mice were subjected to immunohistochemical staining for the proteins β-catenin, Lef1, HO-1, and GPX4. Scale bar: 50 μm. **G** The expression levels of β-catenin, xCT, Lef1, HO-1, and GPX4 proteins were assessed in the tissues obtained from mice after treatment. **H** A diagram illustrating the proposed mechanism

To verify the inhibitory effect of Fe-CDs on the proliferation and invasion of B16 cells and elucidate the underlying mechanism, whole transcriptome mRNA sequencing was performed on tumor tissues treated with Fe-CDs. The sequencing results showed that a total of 230 genes were differentially expressed between Fe-CDs and the control group (p < 0.05), of which 136 genes were up-regulated (e.g., FOXO1, MAPK1, CAMK2A, HMOX1) and 94 genes were down-regulated (e.g., Lef1, FZD10, Wnt11b, Slc7a11, Slc3a2) (Fig. 7C; Additional file 1: Fig. S17). Kyoto Encyclopedia of Genes and Genomes (KEGG) of biological functions of genes found that up-regulated genes in the Fe-CDs group were enriched in pathways promoting apoptosis and DNA damage repair, with representative signaling pathways included ferroptosis pathway (Fig. 7A) and Wnt pathway (Fig. 7B). Further analysis of these two representative signaling pathways identified related genes, including Slc7a11 and Lef1, which were significantly downregulated in the Fe-CDs group. Previous studies have found that the over-expression of Slc7a11 in human tumor cells can inhibit ROS-induced ferroptosis and attenuate the p53-mediated suppression of tumor growth [58, 59]. Here, the levels of Slc7a11 and Slc3a2 genes were decreased in the Fe-CDs group, which resulted in the decrease of GSH synthesis, the indirectly reduction of cell antioxidant capacity, and the enhancement of the cell sensitivity of towards ferroptosis (Fig. 7D). In recent years, a variety of interacting components and functions have been identified in the Wnt signaling pathway, revealing not only the complexity but also the potential of Wnt signaling. It is currently believed that the canonical Wnt/β-catenin signaling pathway is involved in the promotion of cancer initiation and progression [60]. Gene set enrichment analysis (GSEA) was conducted on the significantly differentially expressed genes in melanogenesis, revealing a downregulation in the expression of transcriptional regulators associated with the WNT pathway (Fig. 7E). As a downstream molecule of the Wnt pathway, β-catenin is present inside and outside the cells, and contributes to the adhesion of the trans-membrane adhesion protein VE cadherin to the cell membrane, thereby increasing the stability of the cytoskeleton, supporting angiogenic processes such as vessel sprouting and elongation, and promoting vessel maturation. The Fe-CDs treatment could significantly down-regulate FZD10 and Lef1 in B16 cells, thereby inhibiting the expression of Wnt signaling pathway, inducing apoptosis and preventing proliferation of B16 cells, and playing a certain role in anti-tumor growth and anti-metastasis. To validate the accuracy of the sequencing analysis results, we conducted immunohistochemistry (IHC) experiments on the tumor tissue after treatments, which demonstrated that the expression levels of key genes associated with the Wnt pathway decreased in the Fe-CDs and Fe-CDs@Ce6 + PDT groups, such as β-catenin and Lef1. Simultaneously, genes related to ferroptosis, notably GPX4 and HO-1, showed significantly decreased and increased expression levels, respectively (Fig. 7F; Additional file 1: Fig. S18). It is reported that elevated HO-1 levels can promote the generation of divalent iron ions, thereby facilitating ferroptosis [61]. Furthermore, WB results also indicated that the expression levels of β-catenin and Lef1 decreased, which proved the IHC results. Additionally, genes related to ferroptosis (xCT and GPX4) exhibited reduced expression, while HO-1 expression increased in the Fe-CDs, Fe-CDs@Ce6 and Fe-CDs@Ce6 + PDT groups (Fig. 7G; Additional file 1: Fig. S19), which further confirmed that Fe-CDs could exert anti-tumor effects by promoting ferroptosis. In summary, Fe-CDs had revealed their molecular mechanisms through whole transcriptome sequencing by inhibiting melanoma cell proliferation, activating ferroptosis pathways, and suppressing the Wnt pathway (Fig. 7H). The multi-functional nanoplatform based on Fe-CDs@Ce6 combined with PDT demonstrated great potential in the treatment and diagnosis of melanoma, which provided promising avenues for future therapeutic research and clinical applications, while also offering valuable insights into deeper understanding of melanoma biology.

## Conclusions

In summary, we successfully demonstrate Fe-CDs@Ce6 nanoconjugates by coupling ferroptosis nanoinducer Fe-CDs with photosensitizer Ce6. Benefiting from the nanoparticle structure, Fe-CDs can efficiently enter cells via endocytosis and self-enrich within melanoma, thus serving as a biocompatible and water soluble nanocarrier for carrying Ce6 molecules. The doping of Fe^3+^ in Fe-CDs can induce GSH-responsive ferroptosis, promoting apoptosis and inhibiting invasion of B16 cells by suppressing the Wnt/β-catenin pathway. Combining Fe-CDs with Ce6 appears to further enhance the therapeutic potential of ferroptosis based on the synergistic enhancement of ROS production. In a mouse orthotopic melanoma model, the tumors after Fe-CDs@Ce6 administration with laser irradiation completely disappeared without toxic side effects in normal tissues and organs. As a result of loading iron ions and chlorin, Fe-CDs@Ce6 also exhibits the capability of *T*_*2*_-weighted MRI and FL dual-modal tumor imaging, indicating its potential in imaging-guided tumor therapy. The reported Fe-CDs@Ce6 not only show application prospects in treatment of melanoma but also extensive possibility in other types of tumor, such as breast cancer, lung cancer and brain cancer. In particular, the multifunctionality and specificity of Fe-CDs endow their prominent advantages in loading other moieties and further integrating therapies, which provides a promising research direction in the construction of nanoplatform for cancer therapy.

### Supplementary Information

**Additional file 1: Figure S1.** PL spectra of Fe-CDs under different excitation wavelengths. **Figure S2.** Deconvoluted C 1s and O 1s XPS spectra of Fe-CDs. **Figure S3.** FTIR spectrum of Fe-CDs and Fe-CDs@Ce6. **Figure S4.** XPS full spectra, deconvoluted C 1s and O 1s spectra of Fe-CDs@Ce6. **Figure S5.** Evaluation of stability of Fe-CDs@Ce6 (2 mg/mL) in PBS (pH=7.4), acidic PBS (pH=5.0) and serum (10% FBS). **Figure S6.** Photographs of solution for the chromatic reaction of Fe ions and SA. From left to right: SA, SA+FeSO_4_, SA+Fe-CDs (0.2 mg/mL), SA+Fe-CDs (0.5 mg/mL), SA+GSH-reduced Fe-CDs (0.2 mg/mL), and SA+GSH-reduced Fe-CDs (0.5 mg/mL). **Figure. S7** Photographs of solution for the chromogenic reaction of Fe^2+^ and K_3_[Fe(CN)_6_]. From left to right: (a) 0.1 mg/mL K_3_[Fe(CN)_6_], (b) 0.1 mg/mL K_3_[Fe(CN)_6_] + 0.01 mg/mL FeSO_4_, (c) 0.1 mg/mL K_3_[Fe(CN)_6_] + 0.01 mg/mL Fe_2_(SO_4_)_3_, (d) 0.1 mg/mL K_3_[Fe(CN)_6_] + 0.1 mg/mL Fe-CDs, (e) 0.1 mg/mL K_3_[Fe(CN)_6_] + 0.2 mg/mL Fe-CDs, (f) 0.1 mg/mL K_3_[Fe(CN)_6_] + 0.5 mg/mL Fe-CDs. **Figure S8.** Evaluation of Fenton reaction by measuring the UV-Vis absorption spectra of MB and H_2_O_2_ under different concentrations of Fe-CDs (A) and GSH reduced Fe-CDs (B) (insets show the solution photographs of MB+H_2_O_2_ and different concentrations of Fe-CDs and GSH reduced Fe-CDs). **Figure. S9** ELISA assay of TNF-α (A) and IL-10 (B) expression level in B16 conditioned medium after Fe-CDs@Ce6+PDT treatment. **Figure S10.** **A-B** The levels of ROS in B16 cells after treatment with Fe-CDs (Scale bar: 500 μm). **C-D** The alteration of mitochondrial membrane potential in B16 cells after treatment with Fe-CDs (Scale bar：100 μm) (n=3, **p<0.01, ***p<0.001 were considered statistically significant). **Figure S11.** Western blot analysis for β-catenin, xCT, Lef1, HO1 and GPX4 (n=3, *p<0.05, **p<0.01, ***p<0.001 were considered statistically significant). **Figure S12.** Stimulation of tube formation in HUVECs using B16 cell supernatant after treatment (Scale bar: 200 μm and 100 μm from top to bottom) (n=3, ****p<0.0001 were considered statistically significant). **Figure S13.** **A** Macroscopic presentation of tumors, along with measurements of **B** tumor volume and **C** mouse body weight (n=6) (Scale bar: 1 cm). **Figure. S14** Co-staining of Lyso- and Mito tracker with Fe-CDs@Ce6 at the incubation temperature of 4 and 37°C. Scale bar: 100 μm. **Figure. S15** Time-dependent cellular uptake of Fe-CDs@Ce6 by B16 cells at the incubation temperature of 4 and 37°C. Scale bar: 100 μm. **Figure S16.** The result of H&E staining of main organs of nude mice after treatment (Scale bar: 100 μm). **Figure S17.** Volcano plot of melanoma in PBS vs Fe-CDs. **Figure S18.** Immunohistochemical analysis of β-catenin, Lef1, HO1 and GPX4 (n=3, **p<0.01, ***p<0.001, ****p<0.0001 were considered statistically significant). **Figure S19.** Western blot analysis for β-catenin, xCT, Lef1, HO1 and GPX4 (n=3, **p<0.01, ***p<0.001, ****p<0.0001 were considered statistically significant).

## Data Availability

All data generated or analyzed during this study are included in this article and its Additional file.

## Abbreviations

PDT: Photodynamic therapy
CDs: Carbon dots
Ce6: Chlorin e6
Fe-CDs: Fe ions-doped carbon dots
B16: Mouse skin melanoma cells
^1^O_2_: Singlet oxygen
MRI: Magnetic resonance imaging
ROS: Reactive oxygen species
GSH: Glutathione
GPX4: Glutathione peroxidase 4
GSSG: Oxidized glutathione
PS: Photosensitizers
GlcFe: Ferrous gluconate hydrate
DCC: *N*,*N*′-Dicyclohexylcarbodiimide
DMAP: 4-Dimethylaminopyridine
DMSO: Dimethyl sulfoxide
DI: Deionized
TEM: Transmission electron microscope
PL: Photoluminescence
XPS: X-ray photoelectron spectroscopy
FTIR: Fourier transformed infrared spectra
UV–Vis: Ultraviolet–Visible
ICP: Inductively coupled plasma spectrometer
PBS: Phosphate buffered saline
FBS: Fetal bovine serum
HUVECs: Human umbilical vein endothelial cells
ECM: Endothelial cell medium
RSC96: Rat Schwann cells
DMEM: Dulbecco’s modified eagle medium
H&E: Hematoxylin–eosin
IHC: Immunohistochemistry
WB: Western blot
GO: Gene Ontology
KEGG: Kyoto Encyclopedia of Genes and Genomes
qRT-PCR: Real-time quantitative reverse transcription-polymerase chain reaction
MB: Methylene blue
GSEA: Gene set enrichment analysis
