# Supplementary material for: Ce6-modified Fe ions-doped carbon dots as multifunctional nanoplatform for ferroptosis and photodynamic synergistic therapy of melanoma
Source: J Nanobiotechnology. 2024 Mar 10;22:100. doi: 10.1186/s12951-024-02346-2 (PMC10924998; doi:10.1186/s12951-024-02346-2)
Supplement: Supplementary file 1 — Additional file 1: Figure S1. PL spectra of Fe-CDs under different excitation wavelengths. Figure S2. Deconvoluted C 1s and O 1s XPS spectra of Fe-CDs. Figure S3. FTIR spectrum of Fe-CDs and Fe-CDs@Ce6. Figure S4. XPS full spectra, deconvoluted C 1s and O 1s spectra of Fe-CDs@Ce6. Figure S5. Evaluation of stability of Fe-CDs@Ce6 (2 mg/mL) in PBS (pH=7.4), acidic PBS (pH=5.0) and serum (10% FBS). Figure S6. Photographs of solution for the chromatic reaction of Fe ions and SA. From left to right: SA, SA+FeSO4, SA+Fe-CDs (0.2 mg/mL), SA+Fe-CDs (0.5 mg/mL), SA+GSH-reduced Fe-CDs (0.2 mg/mL), and SA+GSH-reduced Fe-CDs (0.5 mg/mL). Figure. S7 Photographs of solution for the chromogenic reaction of Fe2+ and K3[Fe(CN)6]. From left to right: (a) 0.1 mg/mL K3[Fe(CN)6], (b) 0.1 mg/mL K3[Fe(CN)6] + 0.01 mg/mL FeSO4, (c) 0.1 mg/mL K3[Fe(CN)6] + 0.01 mg/mL Fe2(SO4)3, (d) 0.1 mg/mL K3[Fe(CN)6] + 0.1 mg/mL Fe-CDs, (e) 0.1 mg/mL K3[Fe(CN)6] + 0.2 mg/mL Fe-CDs, (f) 0.1 mg/mL K3[Fe(CN)6] + 0.5 mg/mL Fe-CDs. Figure S8. Evaluation of Fenton reaction by measuring the UV-Vis absorption spectra of MB and H2O2 under different concentrations of Fe-CDs (A) and GSH reduced Fe-CDs (B) (insets show the solution photographs of MB+H2O2 and different concentrations of Fe-CDs and GSH reduced Fe-CDs). Figure. S9 ELISA assay of TNF-α (A) and IL-10 (B) expression level in B16 conditioned medium after Fe-CDs@Ce6+PDT treatment. Figure S10. A-B The levels of ROS in B16 cells after treatment with Fe-CDs (Scale bar: 500 μm). C-D The alteration of mitochondrial membrane potential in B16 cells after treatment with Fe-CDs (Scale bar：100 μm) (n=3, **p<0.01, ***p<0.001 were considered statistically significant). Figure S11. Western blot analysis for β-catenin, xCT, Lef1, HO1 and GPX4 (n=3, *p<0.05, **p<0.01, ***p<0.001 were considered statistically significant). Figure S12. Stimulation of tube formation in HUVECs using B16 cell supernatant after treatment (Scale bar: 200 μm and 100 μm from top to bottom) (n=3, * [file 12951_2024_2346_MOESM1_ESM.docx]

Additional Information

**Ce6-modified Fe Ions-doped Carbon Dots as Multifunctional Nanoplatform for Ferroptosis and Photodynamic Synergistic Therapy of Melanoma**

Haiqiu Li^1^†, Yichen Dou^1^†, Hang Yang^2^, Hanlin Xing^1^, Cheng Zhu^1^, Tao Wang^1^*, Zhaopeng Xuan^1^*, Mingxi Yang^1,3^*

* Corresponding Authors: Tao Wang, Zhaopeng Xuan, Mingxi Yang

[zhhx1974@jlu.edu.cn;](mailto:zhhx1974@jlu.edu.cn;) [xuanzp@mails.jlu.edu.cn;](mailto:jluldw@jlu.edu.cn;) [yangmxchem@jlu.edu.cn](mailto:byangchem@jlu.edu.cn)

**Additional Figures**


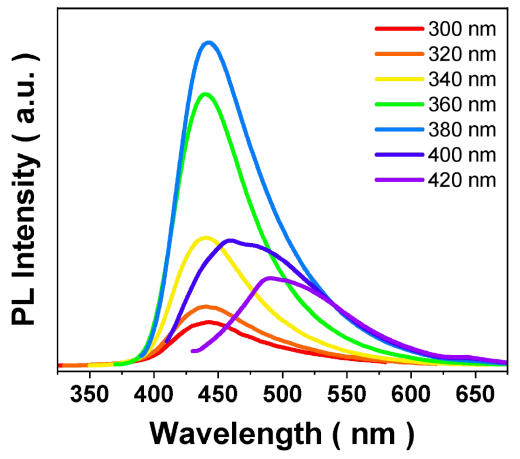


**Figure S1.** PL spectra of Fe-CDs under different excitation wavelengths.


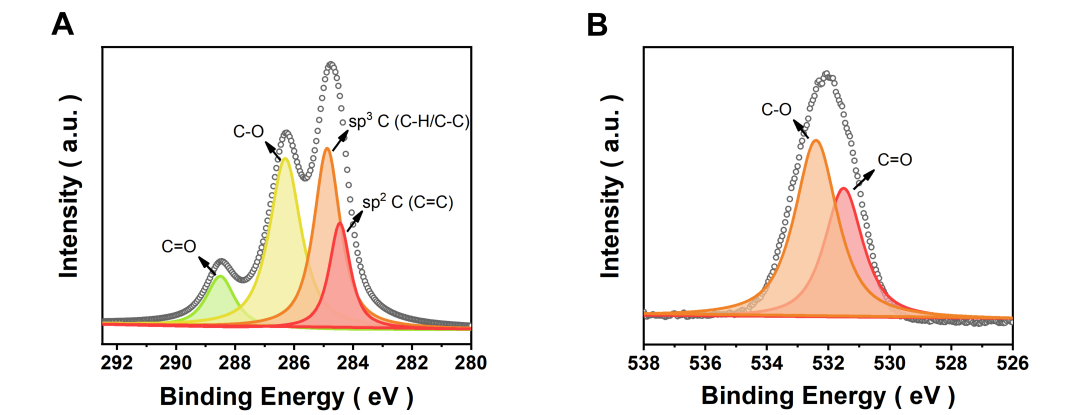


**Figure S2.** Deconvoluted C 1s and O 1s XPS spectra of Fe-CDs.


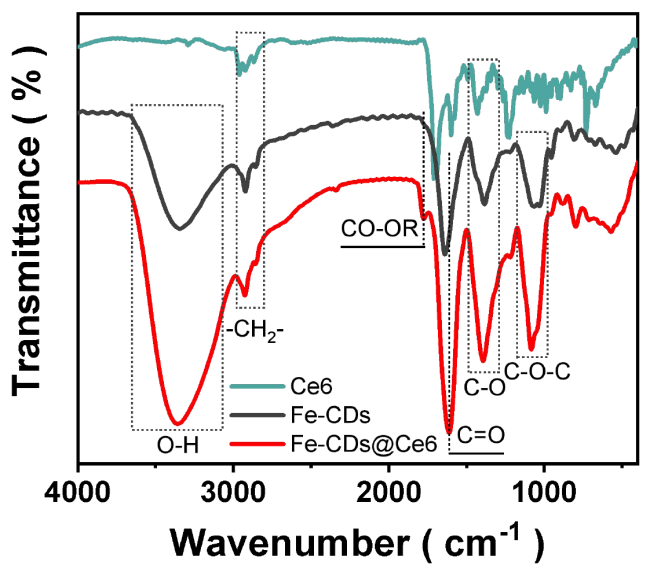


**Figure S3.** FTIR spectrum of Fe-CDs and Fe-CDs@Ce6.


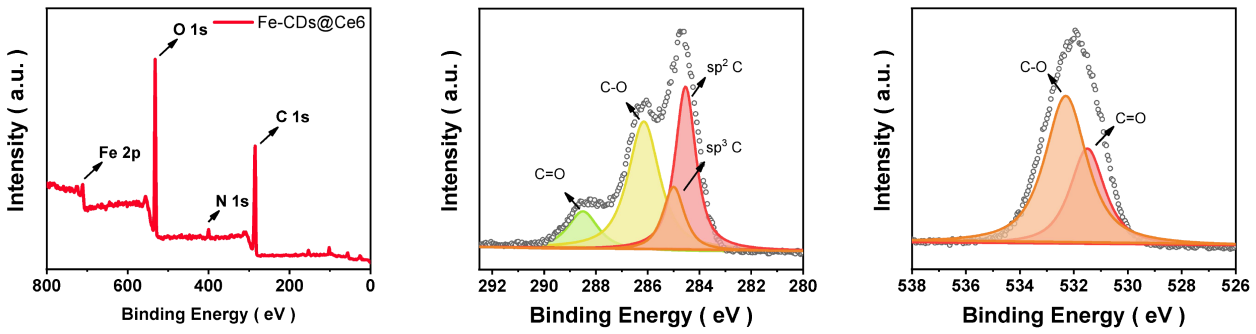


**Figure S4.** XPS full spectra, deconvoluted C 1s and O 1s spectra of Fe-CDs@Ce6.


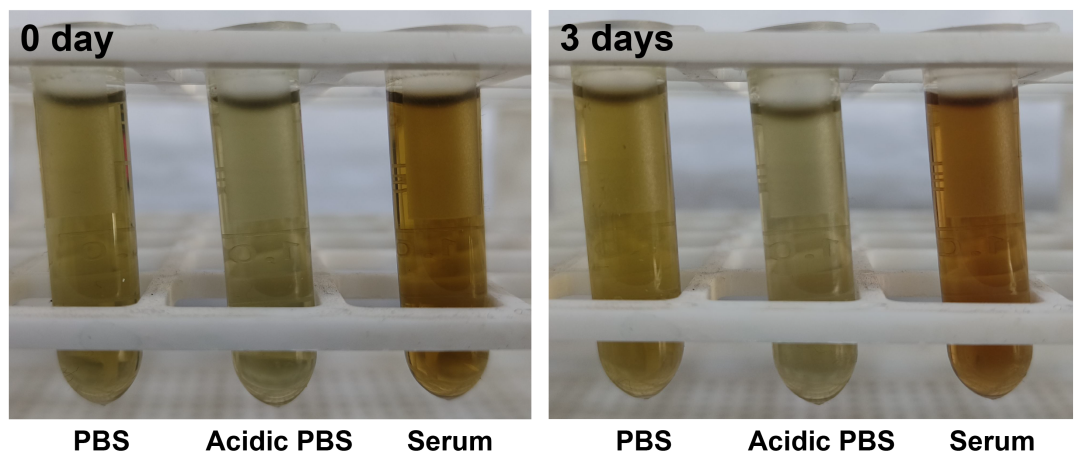


**Figure S5.** Evaluation of stability of Fe-CDs@Ce6 (2 mg/mL) in PBS (pH=7.4), acidic PBS (pH=5.0) and serum (10% FBS).


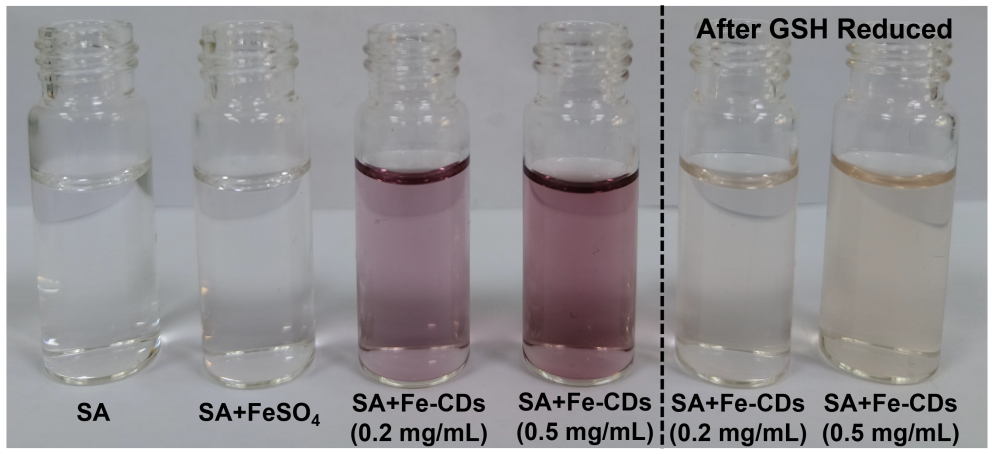


**Figure S6.** Photographs of solution for the chromatic reaction of Fe ions and SA. From left to right: SA, SA+FeSO_4_, SA+Fe-CDs (0.2 mg/mL), SA+Fe-CDs (0.5 mg/mL), SA+GSH-reduced Fe-CDs (0.2 mg/mL), and SA+GSH-reduced Fe-CDs (0.5 mg/mL).

**
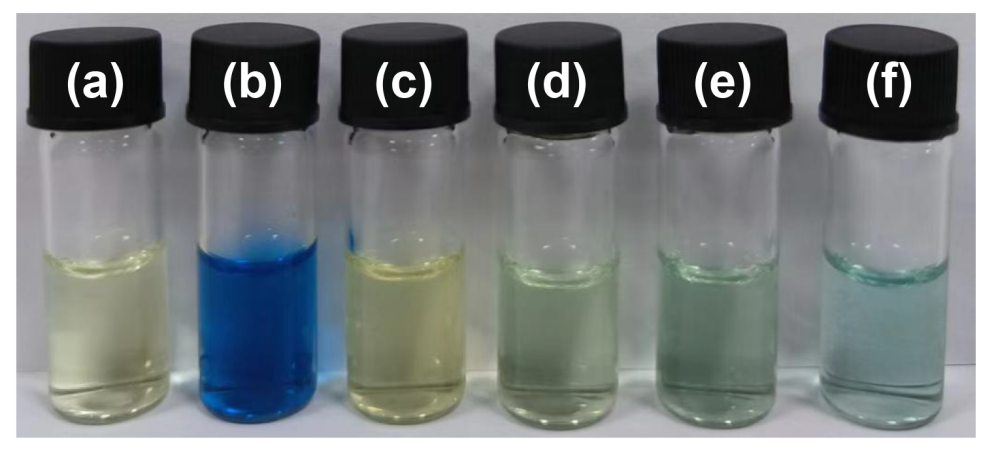
**

**Figure. S7** Photographs of solution for the chromogenic reaction of Fe^2+^ and K_3_[Fe(CN)_6_]. From left to right: (a) 0.1 mg/mL K_3_[Fe(CN)_6_], (b) 0.1 mg/mL K_3_[Fe(CN)_6_] + 0.01 mg/mL FeSO_4_, (c) 0.1 mg/mL K_3_[Fe(CN)_6_] + 0.01 mg/mL Fe_2_(SO_4_)_3_, (d) 0.1 mg/mL K_3_[Fe(CN)_6_] + 0.1 mg/mL Fe-CDs, (e) 0.1 mg/mL K_3_[Fe(CN)_6_] + 0.2 mg/mL Fe-CDs, (f) 0.1 mg/mL K_3_[Fe(CN)_6_] + 0.5 mg/mL Fe-CDs.


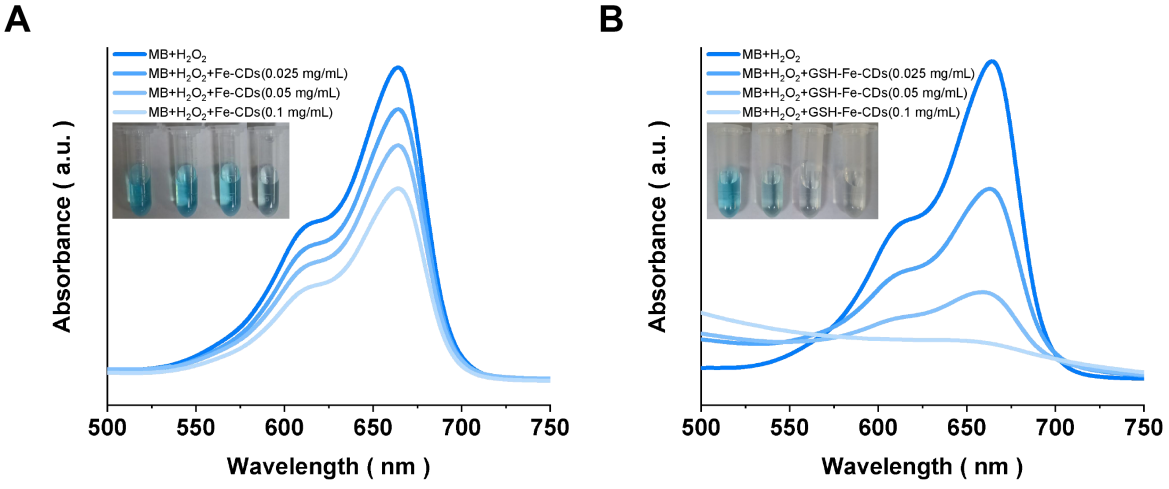


**Figure S8.** Evaluation of Fenton reaction by measuring the UV-Vis absorption spectra of MB and H_2_O_2_ under different concentrations of Fe-CDs (A) and GSH reduced Fe-CDs (B) (insets show the solution photographs of MB+H_2_O_2_ and different concentrations of Fe-CDs and GSH reduced Fe-CDs).


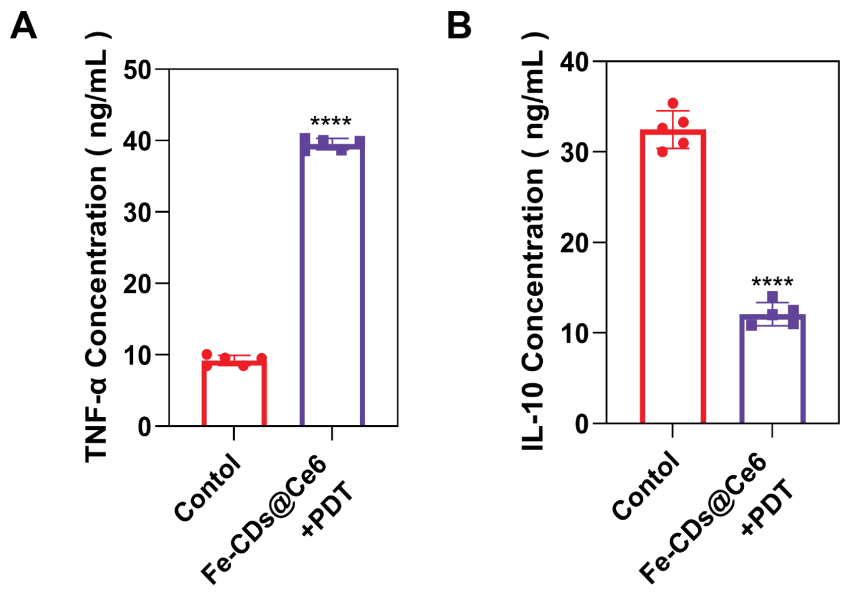


**Figure. S9** ELISA assay of TNF-α (A) and IL-10 (B) expression level in B16 conditioned medium after Fe-CDs@Ce6+PDT treatment.


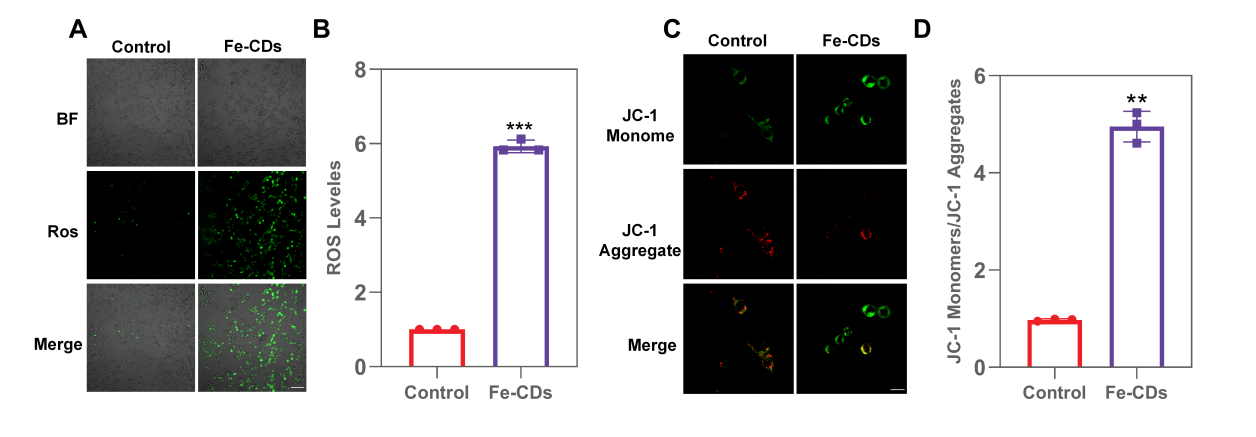


**Figure S10.** **A-B** The levels of ROS in B16 cells after treatment with Fe-CDs (Scale bar: 500 μm). **C-D** The alteration of mitochondrial membrane potential in B16 cells after treatment with Fe-CDs (Scale bar：100 μm) (n=3, **p<0.01, ***p<0.001 were considered statistically significant).


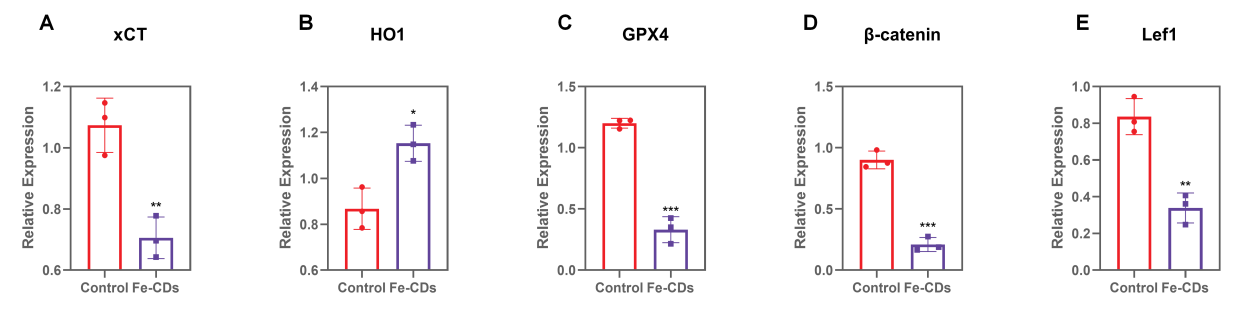


**Figure S11.** Western blot analysis for β-catenin, xCT, Lef1, HO1 and GPX4 (n=3, *p<0.05, **p<0.01, ***p<0.001 were considered statistically significant).


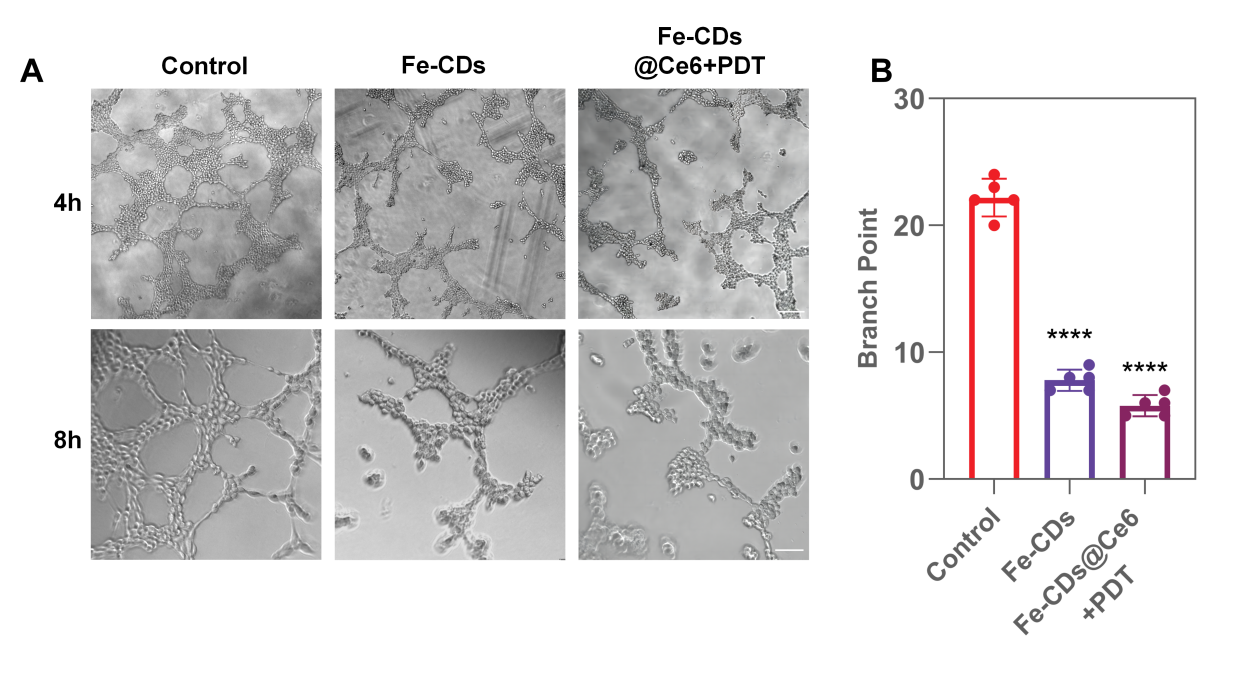


**Figure S12.** Stimulation of tube formation in HUVECs using B16 cell supernatant after treatment (Scale bar: 200 μm and 100 μm from top to bottom) (n=3, ****p<0.0001 were considered statistically significant).


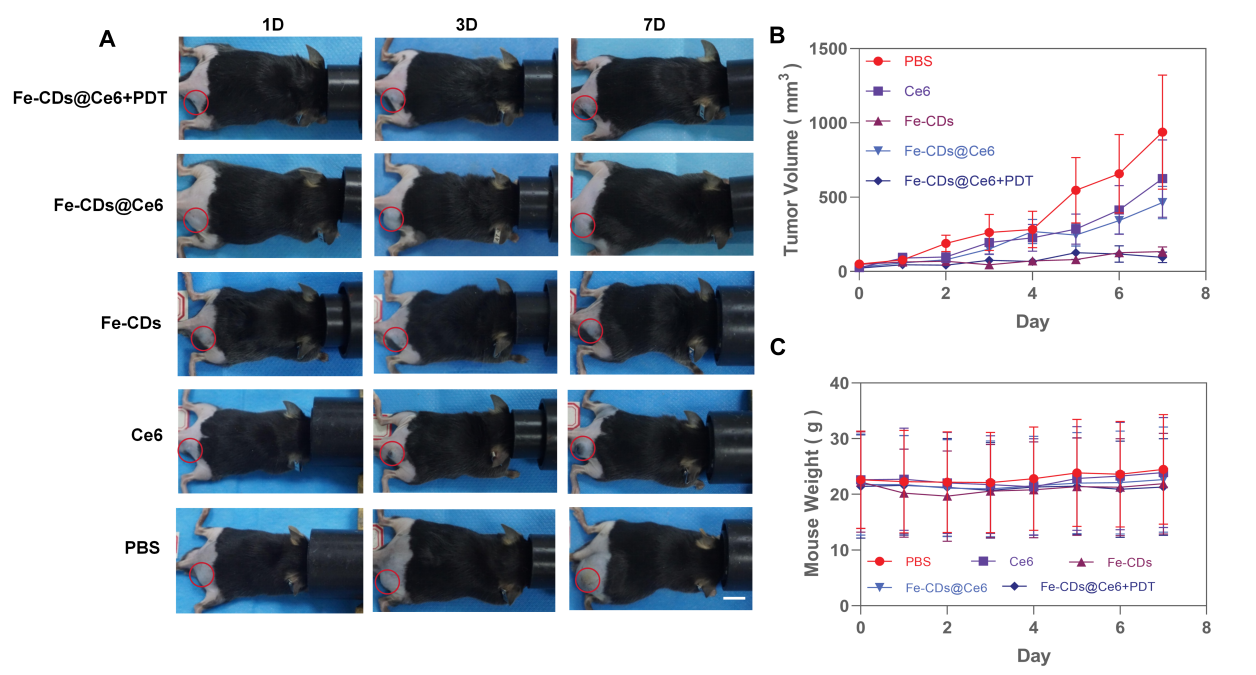


**Figure S13.** **A** Macroscopic presentation of tumors, along with measurements of **B** tumor volume and **C** mouse body weight (n=6) (Scale bar: 1 cm).


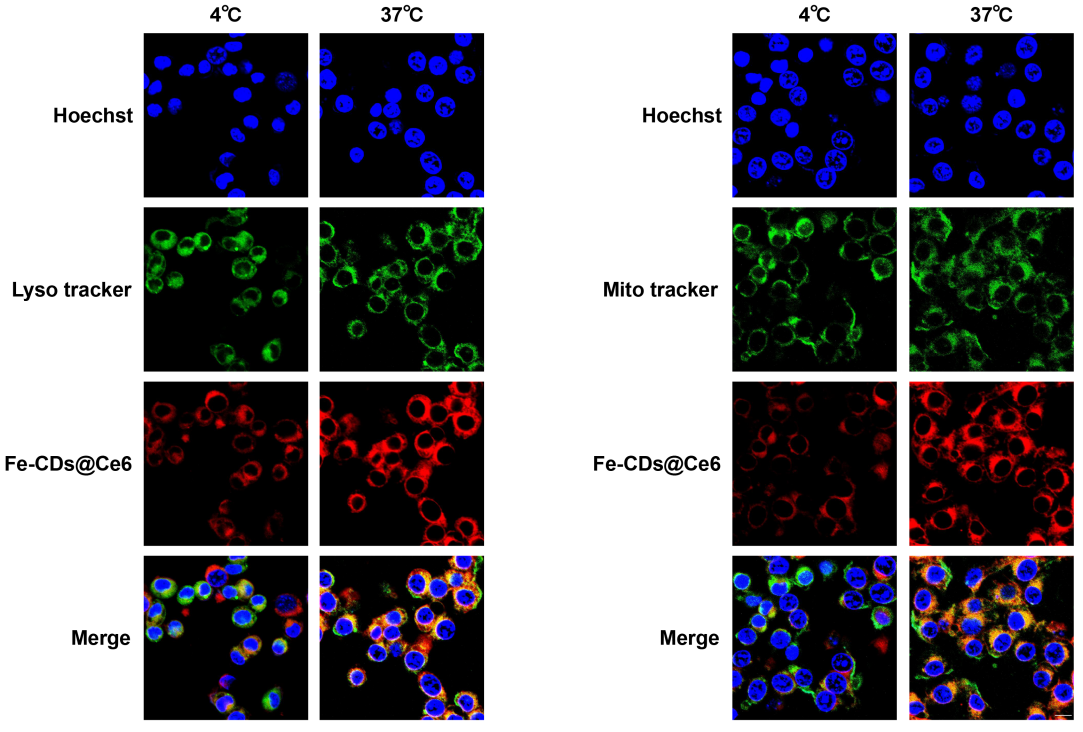


**Figure. S14** Co-staining of Lyso- and Mito tracker with Fe-CDs@Ce6 at the incubation temperature of 4 and 37°C. Scale bar: 100 μm.


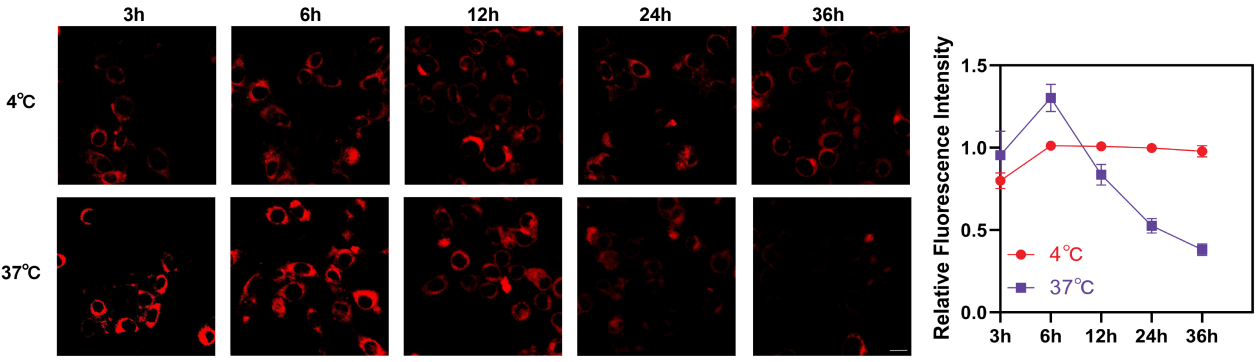


**Figure. S15** Time-dependent cellular uptake of Fe-CDs@Ce6 by B16 cells at the incubation temperature of 4 and 37°C. Scale bar: 100 μm.


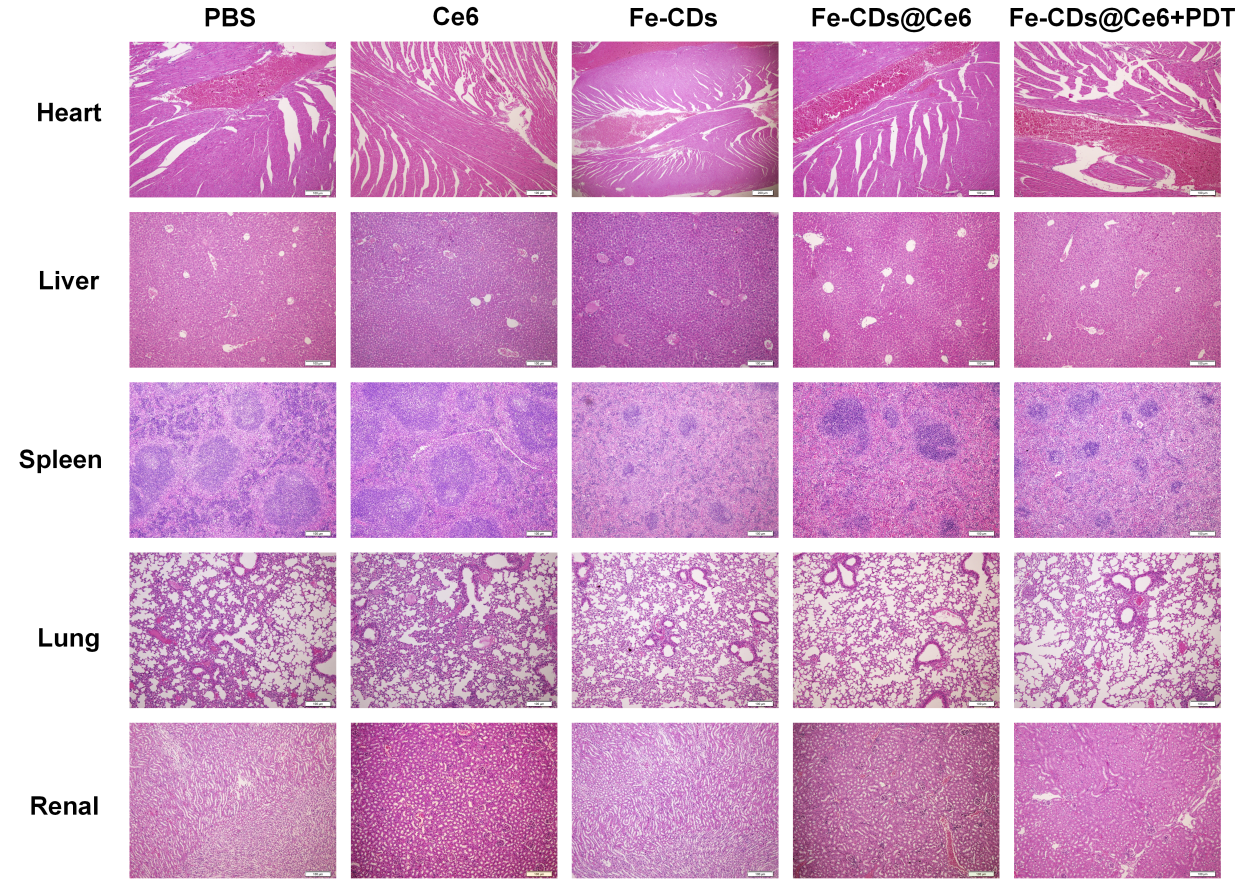


**Figure S16.** The result of H&E staining of main organs of nude mice after treatment (Scale bar: 100 μm).


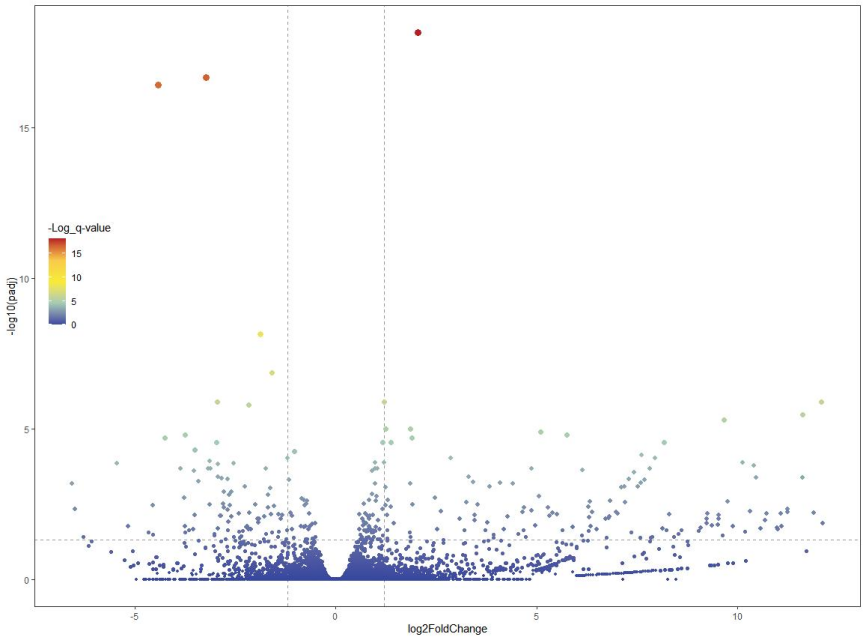


**Figure S17.** Volcano plot of melanoma in PBS vs Fe-CDs.


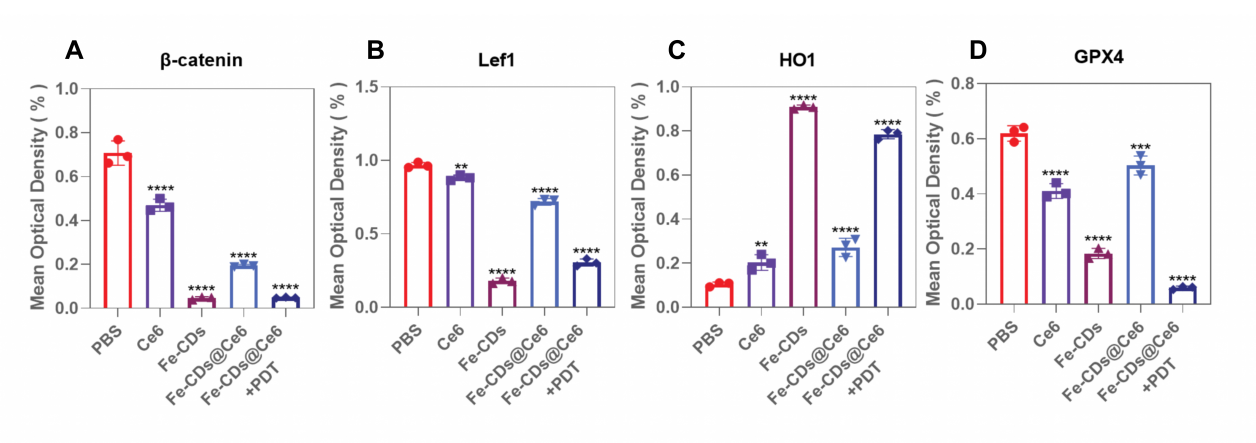
**Figure S18.** Immunohistochemical analysis of β-catenin, Lef1, HO1 and GPX4 (n=3, **p<0.01, ***p<0.001, ****p<0.0001 were considered statistically significant).


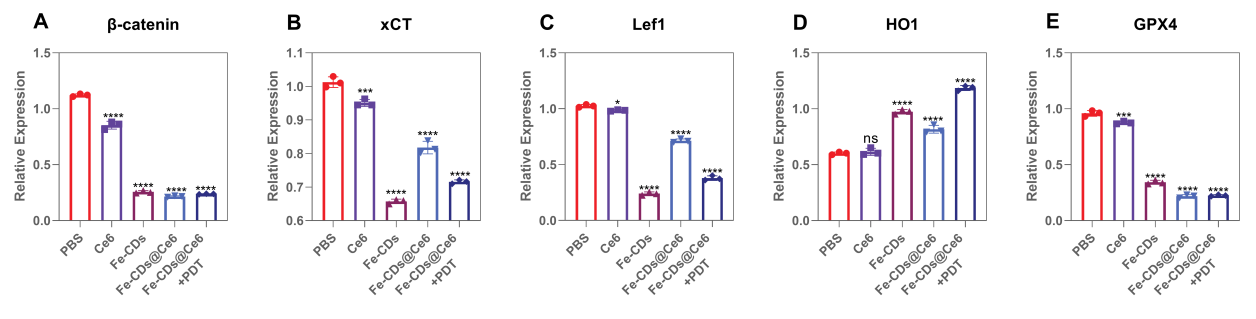


**Figure S19.** Western blot analysis for β-catenin, xCT, Lef1, HO1 and GPX4 (n=3, **p<0.01, ***p<0.001, ****p<0.0001 were considered statistically significant).
